# A functional genetic screen for metabolic proteins unveils GART and the *de novo* purine biosynthetic pathway as novel targets for the treatment of luminal A ERα expressing primary and metastatic invasive ductal carcinoma

**DOI:** 10.3389/fendo.2023.1129162

**Published:** 2023-04-18

**Authors:** Manuela Cipolletti, Stefano Leone, Stefania Bartoloni, Filippo Acconcia

**Affiliations:** Department of Sciences, Section Biomedical Sciences, and Technology, University Roma Tre, Rome, Italy

**Keywords:** breast cancer, estrogen receptor α, 17β-estradiol, lometrexol, GART, *de novo* purine biosynthetic pathway, metabolic reprogramming

## Abstract

Targeting tumor cell metabolism is a new frontier in cancer management. Thus, metabolic pathway inhibitors could be used as anti-estrogen receptor α (ERα) breast cancer (BC) drugs. Here, the interplay among metabolic enzyme(s), the ERα levels and cell proliferation was studied. siRNA-based screen directed against different metabolic proteins in MCF10a, MCF-7 and MCF-7 cells genetically resistant to endocrine therapy (ET) drugs and metabolomic analyses in numerous BC cell lines unveil that the inhibition of GART, a key enzyme in the purine *de novo* biosynthetic pathway, induces ERα degradation and prevent BC cell proliferation. We report here that a reduced GART expression correlates with a longer relapse-free-survival (RFS) in women with ERα-positive BCs. ERα-expressing luminal A invasive ductal carcinomas (IDCs) are sensitive to GART inhibition and GART expression is increased in receptor-positive IDCs of high grade and stage and plays a role in the development of ET resistance. Accordingly, GART inhibition reduces ERα stability and cell proliferation in IDC luminal A cells where it deregulates 17β-estradiol (E2):ERα signaling to cell proliferation. Moreover, the GART inhibitor lometrexol (LMX) and drugs approved for clinical treatment of primary and metastatic BC (4OH-tamoxifen and the CDK4/CDK6 inhibitors) exert synergic antiproliferative effects in BC cells. In conclusion, GART inhibition by LMX or other inhibitors of the *de novo* purine biosynthetic pathway could be a novel effective strategy for the treatment of primary and metastatic BCs.

## Background

Tumor growth and metastasis require cancer cells to utilize energy and biomass to sustain their proliferation. This neoplastic transformation occurs due to the simultaneous deregulation of proliferative signals and metabolic reprogramming, which is a well-established hallmark of cancer (1–3). Cancer cells undergo a comprehensive rearrangement of pathways controlling the metabolism of glucose, amino acids, fatty acids, and nucleotides to fulfill the energetic and material requirements necessary for uncontrolled cell proliferation (3, 4). Nonetheless, the extensive reorganization of tumor metabolism inherently harbors a vulnerability, which can render cancer cells potentially susceptible to the inhibition of various metabolic pathways (4). As a result, drugs that target various metabolic enzymes, such as dihydrofolate reductase (DHFR) and fatty acid synthase (FASN), have either been approved for clinical use or are currently in clinical trials for the treatment of various solid tumors, including colorectal, gastric, breast, and lung cancer (4).

Breast cancer (BC) continues to be a lethal disease among women worldwide. At the time of diagnosis, BCs are classified based on 5 clinicopathological surrogates: luminal A (LumA), luminal B (LumB), epidermal growth factor receptor ERBB2/HER2-overexpressing (HER2+), basal epithelial-like (BL), and normal-like (NL). These surrogates can be further classified based on the histological type of the tumor (e.g., invasive ductal carcinoma - IDC, adenocarcinoma - AC), as well as the expression of estrogen receptor α (ERα). LumA and LumB tumors are ERα-positive, whereas all other surrogates are ERα-negative, but they can still be classified according to the histological type of tumor (e.g., invasive ductal carcinoma, adenocarcinoma, papillary carcinoma, etc.). As a result, the clinical classification of BC reflects a range of molecular phenotypes that correspond to specific types of BC. However, luminal tumors expressing ERα and identified as invasive ductal carcinoma (IDC) represent the majority of BC at the time of diagnosis and tend to have a more favorable prognosis compared to breast tumors that do not express ERα (5–8).

Breast cancer (BC) treatment relies on the expression of ERα to implement endocrine therapy (ET), which targets various aspects of 17β-estradiol (E2):ERα signaling to inhibit cell proliferation and limit tumor progression. Aromatase inhibitors (AIs) reduce the amount of circulating hormone by inhibiting E2 production. Selective estrogen receptor modulators (SERMs), such as 4OH-Tamoxifen (Tam), bind the receptor and inhibit its transcriptional activity, while selective estrogen receptor down-regulators (SERDs), such as fulvestrant, bind ERα and induce its 26S proteasome-dependent degradation (5–8). Although ERα-positive BC has different phenotypes, not all of them respond equally to ET drugs. Tam is the mainstay clinical treatment for LumA, while LumB tumors require a combination of both ET and traditional chemotherapy (8, 9). Even though they have been proven effective, resistance to ET drugs, such as aromatase inhibitors or Tam, can occur. Metastatic disease in distant organs, such as the lung, bone, brain, and liver, develops in about 50% of women with luminal BC who receive ET treatment. Metastatic BC (MBC) cells, which still express the ERα, do not respond to ET drugs and are almost impossible to treat, resulting in the death of most patients (5–9).

Thus, in order to effectively treat MBC and prevent the development of resistance to ET, it is essential to take into account the phenotypic heterogeneity of BC and adopt a personalized approach to disease management. This involves identifying novel drug targets and potential drugs specific to each patient for the treatment of BC.

Using our screening platform, which measures various aspects of ERα signaling and cell proliferation simultaneously, we have shown that drugs capable of inducing the degradation of ERα, rather than directly binding to it, can effectively prevent BC cell proliferation. This discovery has led us to identify several FDA-approved drugs, originally designed for other purposes, that exhibit ‘anti-estrogen-like’ properties and can induce ERα degradation, effectively blocking the proliferation of various BC cell lines (10–17).

Recent findings suggest that inhibiting a metabolic enzyme can have a significant impact on the growth and proliferation of breast cancer cells. For instance, inhibiting FASN can hinder the growth and survival of BC cells stimulated by E2, while the acid ceramidase inhibitor ceranib-2 can cause ERα downregulation and apoptosis in BC cells. Additionally, our research shows that methotrexate, a DHFR inhibitor, can induce receptor degradation and anti-proliferative effects in primary and MBC cells that are resistant to Tam (17–19).

Therefore, here, we aimed to investigate whether certain metabolic pathways could regulate receptor stability and the growth of BC cells by conducting experiments with a small siRNA library targeting approximately 140 metabolic proteins from various pathways. We utilized cell lines representing the non-transformed (i.e., MCF10a cells) (20), primary (i.e., MCF-7 cells) (21, 22) and ET resistant metastatic (*i.e.*, CRISPR-Cas9 genome-edited Y537S-mutated ERα expressing cells) phenotypes (23).

The findings indicate that decreasing the levels of GART (phosphoribosylglycinamide formyltransferase, phosphoribosylglycinamide synthetase, phosphoribosylaminoimidazole synthetase), an enzyme that regulates the *de novo* purine biosynthetic pathway, leads to the degradation of ERα and inhibits the growth of BC cells. This effect is specific to BC cells and does not affect the proliferation of normal breast cells.

## Methods

### Cell culture and reagents

MCF10a, MCF-7, T47D-1, ZR-75-1, HCC1428, BT-474, MDA-MB-361, were purchased by ATCC (USA), while EFM192C were purchased by DSMZ (Braunschweig, Germany) and maintained according to the manufacturer’s instructions. 17β-estradiol (E2), DMEM (with and without phenol red), and fetal calf serum were purchased from Sigma-Aldrich (St. Louis, MO). Bradford protein assay kit as well as anti-mouse and anti-rabbit secondary antibodies were obtained from Bio-Rad (Hercules, CA). Antibodies against ERα (F-10, mouse), pS2 (FL-84, rabbit), Bcl-2 (C2 mouse), cyclin D1 (H-295 rabbit), p62^SQSTM^ (D-3 mouse), and cathepsin D (H75 rabbit) were obtained from Santa Cruz Biotechnology (Santa Cruz, CA, USA); anti-GART antibody (ab187716) was purchased by Abcam (Cambridge, UK). Anti-phospho ERα (Ser118, mouse) antibody was obtained from Cell Signaling; anti-vinculin (mouse) and anti-LC3 (mouse) antibodies were purchased from Sigma-Aldrich (St. Louis, MO, USA). Chemiluminescence reagent for Western blot was obtained from BioRad Laboratories (Hercules, CA, USA). Fulvestrant (*i.e.*, ICI182,780) was purchased by Tocris (USA); 4OH-Tamoxifen, cycloheximide (CHX), lometrexol (LMX) and esiRNA library were purchased from Sigma-Aldrich (St. Louis, MO, USA). Palbociclib and abemaciclib were purchased by Selleck Chemicals (USA). PolarScreen™ ERα Competitor Assay Kit, Green (A15882) was acquired from Thermo Scientific. Tissue arrays were obtained by Biotechne (Minneapolis, MN, USA). All the other products were from Sigma-Aldrich. Analytical- or reagent-grade products were used without further purification. The identities of all the used cell lines were verified by STR analysis (BMR Genomics, Italy).

### *In vitro* ERα binding assay

A fluorescence polarization (FP) assay was used to measure the binding affinity of lometrexol (LMX) and 17β-estradiol (E2) for recombinant ERα *in vitro*. The FP assay was performed using a PolarScreen™ ERα Competitor Assay Kit, Green (A15882, Thermo Scientific) as previously reported (24).

### In-cell Western blot

In-cell Western blot was used to measure ERα levels in MCF-7, and Y537S cell lines as previously described (17). The cells were treated with esiRNA targeting different metabolic proteins according to the reverse protocol detailed in (25) in quadruplicate for 48 hours. Fulvestrant (*i.e.*, ICI – 100 nM) was used as the control for ERα degradation.

### In-cell propidium iodide staining

In-cell PI staining was used to measure DNA content in MCF10a, MCF-7, and Y537S cell lines. The experiments were carried out using the protocol previously described (17). The cells were treated with esiRNA targeting different metabolic proteins according to the reverse protocol detailed in (25) in quadruplicate for 48 hours. Taxol (1 µM) was used as the control for cell proliferation.

### Measurement of ERα transcriptional activity

MCF-7 and Y537S cells were stably transfected with a plasmid containing an ERE-nanoluciferase (NLuc)-PEST reporter gene and measurement of NLuc-PEST expression (*i.e.*, ERα transcriptional activity) was performed after 24 hours of compound administration as described (16, 26).

### Cell manipulation for Western blot analyses

Cells were grown in DMEM with phenol red plus 10% fetal calf serum for 24 hours and then treated with the different compounds at the indicated doses for the indicated periods. Before E2 stimulation, cells were grown in DMEM without phenol red plus 10% charcoal-stripped fetal calf serum for 24 hours; lometrexol was added 48 hours before E2 administration. After treatment, cells were lysed in Yoss Yarden (YY) buffer (50 mM Hepes (pH 7.5), 10% glycerol, 150 mM NaCl, 1% Triton X-100, 1 mM EDTA and 1 mM EGTA) plus protease and phosphatase inhibitors. Western blot analysis was performed by loading 20–30 µg of protein on SDS-gels. Gels were run, and the proteins were transferred to nitrocellulose membranes with a Turbo-Blot semidry transfer apparatus from Bio-Rad (Hercules, CA, USA). Immunoblotting was carried out by incubating the membranes with 5% milk or bovine serum albumin (60 min), followed by incubation overnight (o.n.) with the indicated antibodies. Secondary antibody incubation was continued for an additional 60 min. Bands were detected using a Chemidoc apparatus from Bio-Rad (Hercules, CA, USA).

### Tissue array immunohistochemistry

Immunohistochemistry of tissue slides was performed as described in (27). Sections were incubated with the anti-GART antibody described above.

### Small interference RNA

MCF-7 and Y537S cells were transfected with esiRNA against GART and the procedure was carried out using Lipofectamine RNAi Max (Thermo Fisher) as previously reported (25).

### Cell proliferation and 3D cell culture assays

For growth curves, the xCELLigence DP system ACEA Biosciences, Inc. (San Diego, CA) Multi-E-Plate station was used to measure the time-dependent response to the indicated drugs by real-time cell analysis (RTCA), as previously reported (13, 16, 24, 28). Synergy studies and Tam growth curves were done using Crystal Violet staining as reported in (29). Next, the synergy index was calculated with Combenefit freeware software (24). Alginate-based and tumor spheroid cultures were performed and quantitated as previously reported (24).

### RNA isolation and qPCR analysis

The sequences for gene-specific forward and reverse primers were designed using the OligoPerfect Designer software program (Invitrogen, Carlsbad, CA, USA). The primers used for human ERα were 5’-GTGCCTGGCTAGAGATCCTG-3’ (forward) and 5’-AGAGACTTCAGGGTGCTGGA-3’ (reverse), and for human GAPDH were 5’-CGAGATCCCTCCAAAATCAA-3’ (forward) and 5’-TGTGGTCATGAGTCCTTCCA-3’ (reverse). Total RNA was extracted from the cells using TRIzol Reagent (Invitrogen, Carlsbad, CA, USA), according to the manufacturer’s instructions. To determine gene expression levels, cDNA synthesis and qPCR were performed using the GoTaq 2-step RT-qPCR system (Promega, Madison, MA, USA), with an ABI Prism 7900HT Sequence Detection System (Applied Biosystems, Foster City, CA, USA), according to the manufacturer’s instructions. Each sample was tested in triplicates, the experiment was repeated twice, and gene expression was normalized to GAPDH mRNA levels.

### Bromodeoxyuridine incorporation assay

Bromodeoxyuridine (BrdU) was added to the medium in the last 30 min of growth, and the cells were then fixed and permeabilized. Histones were dissociated with 2 M HCl as previously described (30). BrdU-positive cells were detected with anti-BrdU primary antibody diluted 1:100 (DAKO; Santa Clara, CA, USA) and Alexa488-conjugated anti-mouse antibody diluted 1:100 (Thermo Fisher Scientific; Waltham, MA, USA). Both antibodies were incubated with the cells for 1 h at room temperature in the dark. BrdU fluorescence was measured using a CytoFlex flow cytometer, and S-phase analysis was performed with CytExpert v 2.3 software (Beckman Coulter, Brea, CA, USA). All samples were counterstained with propidium iodide (PI) for DNA/BrdU bi-parametric analysis.

### Statistical analysis

Statistical analysis was performed using the InStat version 8 software system (GraphPad Software Inc., San Diego, CA). Densitometric analyses were performed using the freeware software Image J by quantifying the band intensity of the protein of interest with respect to the relative loading control band (*i.e.*, vinculin) intensity. The *p-values* and the used statistical test (i.e., either Student t-test or ANOVA Test) are given in figure captions.

## Results

### Functional metabolic proteins and metabolomic screens identify the *de novo* purine biosynthetic pathway as a potential novel target for ERα-positive BCs

**Figure 1:**
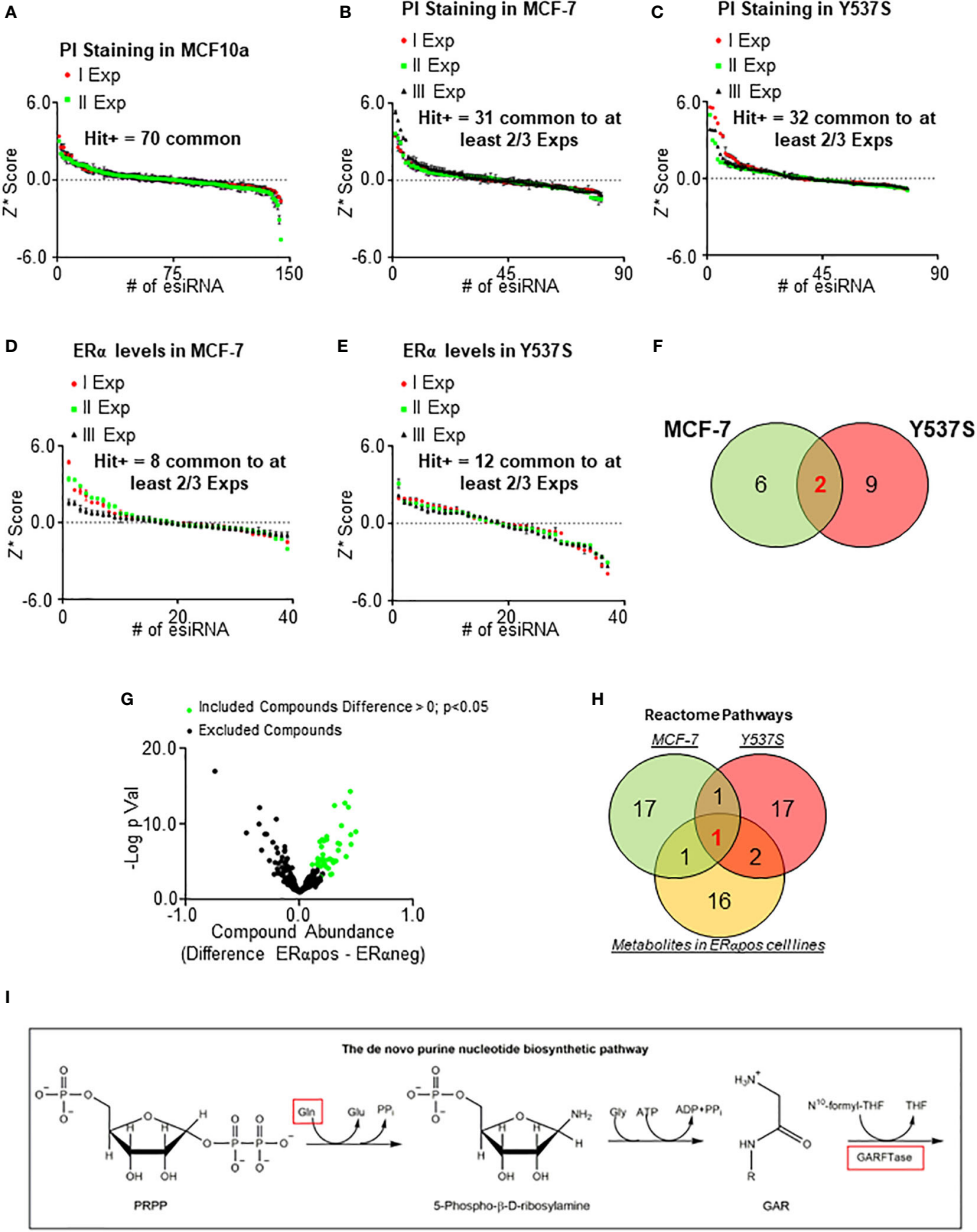
Identification of metabolic pathway influencing ERα levels and cell proliferation in primary and metastatic breast cancer cells. Robust *Z* scores (Z*) graphs for siRNA oligonucleotides (esiRNA)-treated samples for 48 hours in MCF10a **(A)**, MCF-7 **(B, D)**, and Y537S **(C, E)** cells. The ERα levels were detected by in-cell WB **(D, E)** while cell number was detected by in-cell propidium iodide (PI) staining **(A, B, C)**. **(F)** Venn diagrams showing the positive hits for ERα and PI lists in MCF-7 and Y537S cells. **(G)** Volcano plot showing the differences in metabolic compounds among ERα-positive and ERα-negative BC cell lines. Data have been downloaded by the DepMap portal (https://depmap.org/portal). Green dots represent the compound considered as significant according to the thresholds indicated in the figure. **(H)** Venn diagram showing pathway enrichment as extrapolated by the Reactome (https://reactome.org/PathwayBrowser/#/) database for positive hits in either MCF-7 and Y537S cells or metabolites in ERα-positive BC cell lines. **(I)** Schematic showing the initial reaction of the *de novo* purine biosynthetic pathway. Red squares indicate the up-regulated compound in ERα-positive BC cell lines and the enzyme found in both MCF-7 and Y537S cells.

To identify a potential novel target within metabolic proteins that could reduce ERα stability and cell proliferation in BC cells, we reasoned that such protein should be lethal for tumor cells while its inhibition must not significantly affect the proliferation of non-transformed cells. Therefore, we manually collected a library of siRNA directed against about 140 metabolic proteins belonging to different metabolic pathways (i.e., lipid, nucleotide, and amino acid metabolism) and tested it in non-transformed breast cells (i.e., MCF10a cells) (20). Notably, we decided not to include in the library those proteins of the glucose metabolism, as the Warburg effect in the context of cell proliferation has been deeply analyzed (1–4). We measured the ability of siRNA oligonucleotides to reduce cell proliferation by using *in-cell* propidium iodide (PI) staining (14, 17) in MCF10a cells. We calculated the robust *Z* score (Z*) (31, 32) in two different experiments and identified a group of 70 metabolic proteins, which reduction in expression levels did not modify the proliferation of MCF10a cells in both experiments (**Figure 1A**). Next, we transfected these 70 siRNA oligonucleotides in both MFC-7 cells and in MCF-7 cells expressing the Y537S ERα mutant (Y537S) hyperactive receptor variant, which confers resistance to ET (23, 33). The *in-cell* PI staining experiment was performed three times and those siRNA oligonucleotides with a calculated Z* > 0.5 were considered as a positive hit only if such a result was scored in at least 2 out of the 3 experiments. By using these limits, we shortlisted 31 and 32 siRNA oligonucleotides that reduced cell proliferation in MCF-7 and Y537S cells, respectively (**Figures 1B, C**). Next, we applied these 31 and 32 siRNA oligonucleotides in MCF-7 and Y537S cells and measured ERα intracellular levels in *in-cell* Western blot (WB) assays (14, 17). Also in this case we repeated the experiment three times and set a Z* > 0.5 threshold to identify those siRNA oligonucleotides reducing receptor levels. Treatments, which were fished out in at least 2 out of the 3 experiments, were considered positive hits (**Figures 1D, E**). Overall, we identified that the reduction in the expression levels of 8 and 11 metabolic proteins in MCF-7 and Y537S cells, respectively (**Supplementary Table 1**), were potentially able to reduce ERα levels and cell proliferation without affecting the basal growth rate of the non-transformed breast MCF10a cells. Remarkably, 2 of these metabolic proteins were in common between MCF-7 and Y537S cells (**Figure 1F**; **Supplementary Table 1**).

In parallel, we inspected the DepMap portal (https://depmap.org/portal) to understand if the amount of some metabolites was preferentially increased in ERα-positive BC cell lines. The dataset contains the measurement of 225 metabolites in 18 ERα-positive and 30 ERα-negative BC cell lines. For each metabolite, we calculated the mean value in ERα-positive and ERα-negative BC cell lines, then we extrapolated the difference in the metabolite amount between ERα-positive and ERα-negative BC cell lines and finally we generated a p-value (using Student t-test) corresponding to the differences in metabolite abundance between ERα-positive and ERα-negative BC cell lines (**Supplementary Table 2**). The volcano plot in **Figure 1G** shows the -Log_2_ of the p-value as a function of the compound abundance expressed as the difference between ERα-positive and ERα-negative BC cell lines. We considered those metabolites with a difference > 0 and a p-value < 0.05 (green dots in **Figure 1G**; **Supplementary Table 2**) as significantly enriched in ERα-positive versus ERα-negative BC cell lines.

Next, we performed Reactome (https://reactome.org/PathwayBrowser/#/) pathway enrichment analysis both for the metabolic proteins identified in either MCF-7 or Y537S cells (**Figure 1F**; **Supplementary Table 1**) and for the metabolites that are more abundant in ERα-positive than ERα-negative BC cell lines (green dots in **Figure 1G**; **Supplementary Table 2**) and found the *de novo* purine nucleotide biosynthesis as the only common pathway to the 3 datasets (**Figure 1H**).

Accordingly, we found that the abundance of glutamine (**Figure 1I**) (34), a required substrate for *de novo* purine biosynthesis, was higher in ERα-positive than ERα-negative BC cell lines and phosphoribosylglycinamide formyltransferase, phosphoribosylglycinamide synthetase, phosphoribosylaminoimidazole synthetase (GART) (**Figure 1I**) (34), the tripartite enzyme required for the second enzymatic step in the chain of reactions for purine production, was one of the 2 common metabolic proteins, which siRNA-based depletion reduces ERα levels and prevents the proliferation of MCF-7 and Y537S cells.

### The status of the *de novo* purine nucleotide biosynthetic pathway in BC progression

**Figure 2:**
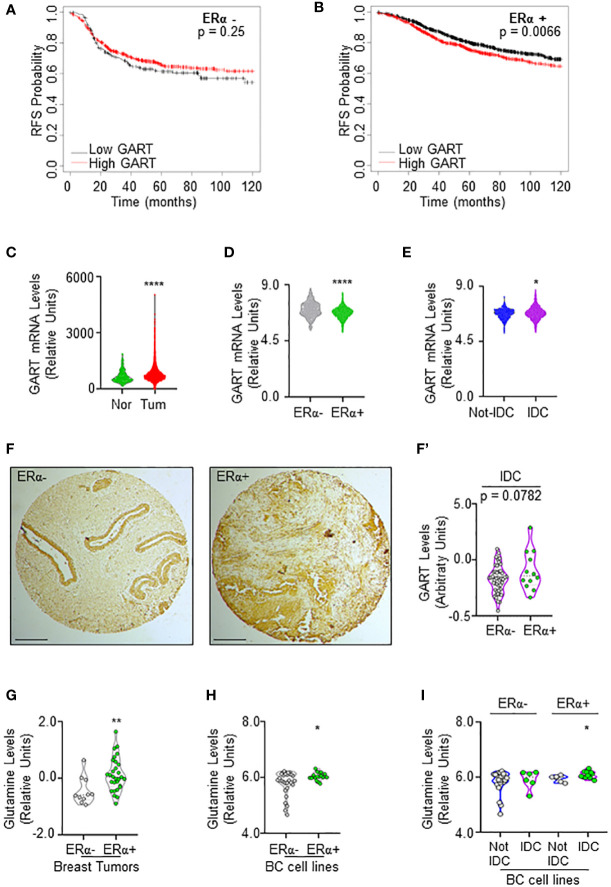
GART and glutamine levels in breast cancer. **(A)** Kaplan-Meier plots showing the relapse-free survival (RFS) probability in women carrying breast tumors expressing either ERα-negative **(A)** or ERα-positive **(B)** BC as a function of GART mRNA levels. Data have been downloaded by the website (https://kmplot.com/analysis/). All possible cutoff values between the lower and upper quartiles are automatically computed (*i.e.*, auto-select best cutoff on the website), and the best performing threshold is used as a cutoff (35). Significant differences between the RFS are given as *p*-value in each panel. **(B)** GART mRNA levels in breast tumors versus normal breast epithelium as downloaded by TNM plot (https://tnmplot.com/analysis/) database (36). **** (*p* < 0.0001) indicates significant differences as calculated by the Student t-test. GART mRNA levels in BC as a function of ERα expression **(D)** or the histological type of the tumor **(E)** downloaded by the cBioPortal (https://www.cbioportal.org/) database. IDC: invasive ductal carcinoma. Not-IDC: other kinds of tumors. Each dot represents one tumor. **** (*p* < 0.0001) indicates significant differences as calculated by the Student t-test. GART immunohistochemical analysis in tissue slides **(F)** and relative quantitation (F’) in invasive ductal carcinomas (IDC) stratified as ERα-negative and ERα-positive. *p-value* for differences is given in the panel. Dots represent the number of the analyzed tumors. **(G)** Glutamine levels in different breast tumors as downloaded by the Breast Cancer landscape (https://www.breastcancerlandscape.org/) (7) and stratified both as ERα-negative or ERα-positive. ** (*p* < 0.01) indicates significant differences as calculated by the Student t-test. Each dot of the plots in **(G)** represents the value of the indicated parameter in a single breast tumor. Glutamine levels in different BC cell lines as downloaded by the DepMap portal (https://depmap.org/portal) and stratified both as ERα-negative or ERα-positive **(H)** and as invasive ductal carcinoma (IDC) or not (Not-IDC) cell lines classified as ERα-negative or ERα-positive **(I)**. * (*p* < 0.05) indicates significant differences as calculated by the Student t-test. Each dot of the plots in **(H, I)** represents the value of the indicated parameter in a single breast cancer cell line.

Prompted by these results, we next evaluated whether different levels of GART mRNA expression could impact the survival of women carrying ERα-negative or ERα-positive BCs. Kaplan-Meier curves were done using the Kaplan-Meier Plotter database (https://kmplot.com/analysis/) (35) and showed that women with ERα-positive BCs expressing low levels of GART have a significantly higher relapse-free survival (RFS) probability with respect to those patients expressing high GART mRNA levels while no changes in RFS rate has been observed in women with ERα-negative BCs (**Figures 2A, B**; **Supplementary Table 4**).

Because these data suggest that GART expression could have a role in ERα-positive BC progression, we next evaluated its mRNA expression in breast tumors versus normal breast epithelium. The data retrieved by the TNM plot (https://tnmplot.com/analysis/) (36) show that breast tumors express higher levels of GART mRNA with respect to their normal counterpart (**Figure 2C**; **Supplementary Table 5**). However, the analysis of the Metabrick datasets extrapolated by the cBioPortal database (https://www.cbioportal.org/) (37, 38) revealed that GART mRNA was slightly but significantly reduced in ERα-positive (i.e., all the luminal tumors) than ERα-negative (i.e., all the other tumors) BCs (**Figure 2D**; **Supplementary Table 6**). To solve the contradiction for which low GART mRNA expression increases the RFS of women carrying ERα-positive tumors but GART mRNA levels are lower in ERα-positive than ERα-negative tumors, we decided to stratify the Metabrick data according to the histological type of the tumor by comparing the invasive ductal carcinoma (IDC - the most frequent one) with all the other types of tumors. As shown in **Figure 2E**; **Supplementary Table 7**, GART mRNA is significantly increased in IDC. Prompted by these results, we next performed immunohistochemical analysis for GART in a set of 74 breast tumors spotted on a tissue array. Quantitation of the amount of GART protein expression in those tumors classified as IDC and stratified as ERα-positive and ERα-negative revealed that ERα-positive IDCs express higher levels of GART than ERα-negative IDCs (**Figures 2F, F’**). Altogether these data indicate that low GART levels are correlated with a higher survival rate in patients with ERα-positive tumors and that GART expression is higher in the IDCs expressing the ERα.

In parallel, because glutamine is one of the upstream substrates of GART in the *de novo* purine biosynthetic pathway (34), we next evaluated if glutamine content was upregulated in the same tumor types by inspecting its abundance both in the breast tumors analyzed in the Breast Cancer proteome, proteogenomic and metabolomics landscape (https://www.breastcancerlandscape.org/) (7) and in the BC cell lines profiled in DepMap portal (https://depmap.org/portal). Glutamine amount was increased in ERα-positive with respect to ERα-negative breast tumors (**Figure 2G**; **Supplementary Table 8**) and BC cell lines (**Figure 2H**; **Supplementary Table 8**). Moreover, the classification of the BC cell lines performed according to the histological type as reported both in Neve et al. (21), and in Dai et al. (22), revealed that ERα-positive IDC cell lines contain an increased level of glutamine than the ERα-positive Not-IDC cell lines while no difference in Not-IDC and IDC cell lines has been detected in glutamine levels among ERα-negative cells (**Figure 2I**; **Supplementary Table 8**).

Overall, these data indicate that both glutamine and GART levels are increased in ERα-positive IDC, thus further suggesting that targeting the *de novo* purine biosynthetic pathway by reducing GART activity could be a valuable strategy to prevent ERα-positive BC progression.

### GART inhibition preferentially affects the proliferation of luminal A ERα-positive invasive ductal carcinoma cells

**Figure 3:**
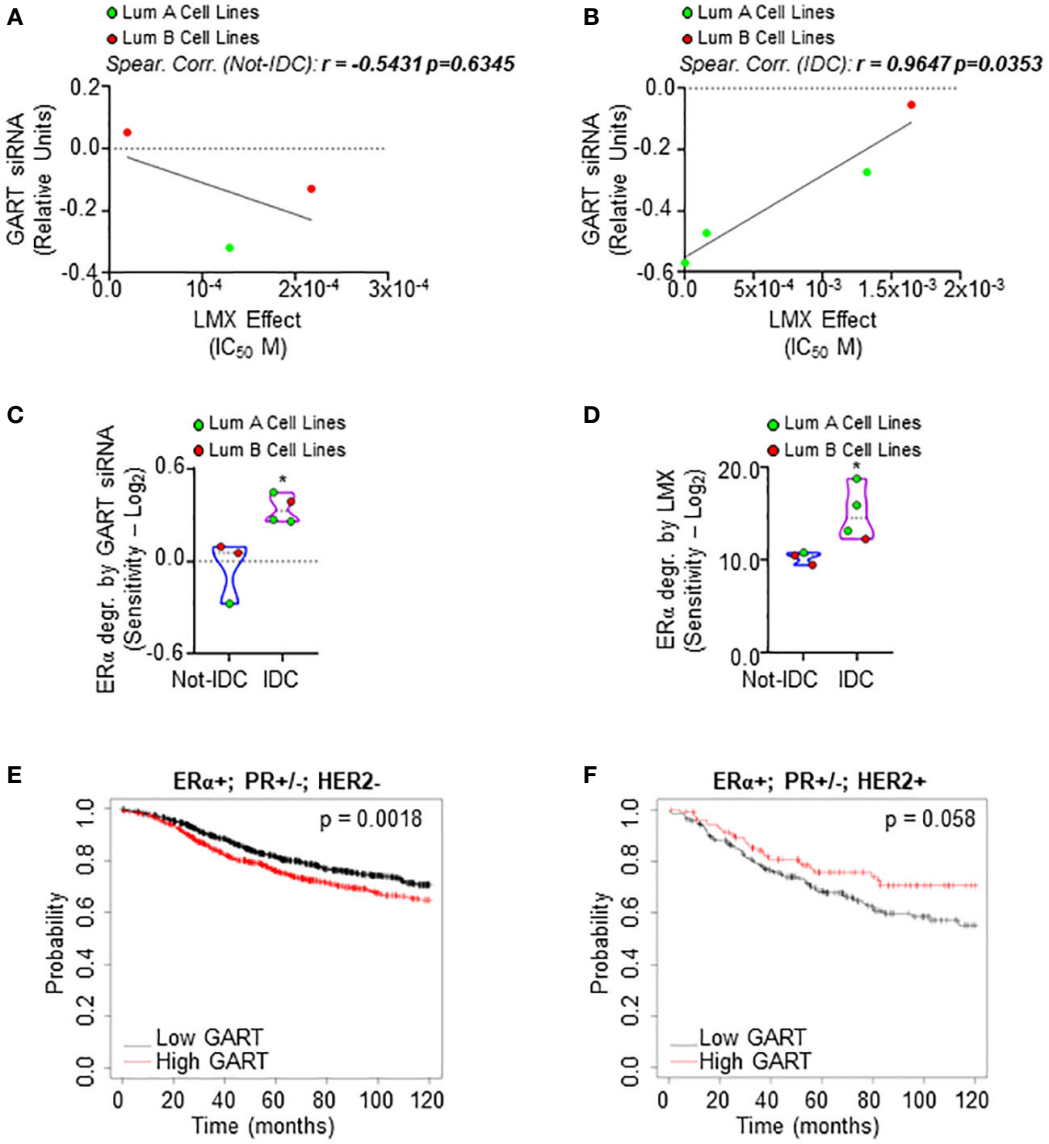
Sensitivity of luminal A ERα-positive invasive ductal carcinomas to GART inhibition. Linear regression and Spearman Correlation values between the sensitivity to GART siRNA-induced anti-proliferative effect as downloaded by the DepMap portal (https://depmap.org/portal) and the calculated inhibitor concentration 50 (IC_50_) for lometrexol (LMX)-induced anti-proliferative effect in the same BC cell lines stratified in luminal A (LumA, green dots) and luminal B (LumB, red dots) BC cell lines and in non-invasive ductal carcinomas (Not-IDC) **(A)** or invasive ductal carcinomas (IDC) **(B)** r and *p-values* are given in the main panels. The sensitivity to GART siRNA-induced **(C)** and lometrexol (LMX)-induced **(D)** ERα degradation effect as calculated by extrapolating the effective concentration 50 (EC_50_) and -Log_2_ transforming it in different BC cell lines stratified as luminal A (LumA, green dots) and luminal B (LumB, red dots) and non-invasive ductal carcinomas (Not-IDC) or invasive ductal carcinomas (IDC). Kaplan-Meier plots showing the relapse-free survival (RFS) probability in women carrying breast tumors expressing ERα, both expressing or not progesterone receptor (PR) but not HER2 **(E)** or ERα, HER2 or both expressing or not PR **(F)** as a function of GART mRNA levels. Significant differences between the RFS are given as *p*-value in each panel. Data have been downloaded by the website (https://kmplot.com/analysis/). All possible cutoff values between the lower and upper quartiles are automatically computed (*i.e.*, auto-select best cutoff on the website), and the best performing threshold is used as a cutoff (35).

Next, we evaluated the effect of both the GART siRNA-dependent downregulation and the GART inhibition by lometrexol (LMX) (39) in a panel of 7 ERα-positive BC cell lines characterized by different histological types and different clinical surrogates (**Supplementary Table 9**). In these 7 cell lines, we compared the siRNA-mediated effect on cell proliferation as retrieved by the DepMap portal database with the inhibitory concentration 50 (IC_50_) at 5 days for the LMX-induced reduction in cell proliferation that we calculated after performing growth curve analyses in the cell lines treated in the presence of different doses of LMX. As shown in **Figures 3A, B**; **Supplementary Table 9**, a significant linear correlation between the antiproliferative effect of GART siRNA and the LMX effect has been evidenced only in IDC cell lines (r = 0.9647 p=0.0353) and not in Not-IDC cells (r = -0.5431 p=0.6345). Interestingly, data also indicate that IDC cell lines belonging to the LumA class of the clinical surrogates are more sensitive to LMX- and GART siRNA-dependent anti-proliferative effect (green dots in **Figure 3B**).

The effect of both the GART siRNA-dependent downregulation and the GART inhibition by LMX on ERα content in the same 7 ERα-positive BC cell lines was further tested. siRNA-mediated experiments and LMX dose-response curves were performed twice in each cell line at 48 hours after siRNA or LMX administration and quantitation of the treatment effect on receptor level [i.e., percentage of reduction for siRNA experiments and effective concentration 50 (EC_50_) for LMX] was -Log_2_ transformed to obtain the sensitivity for each cell line. IDC cell lines are significantly more sensitive to GART siRNA-mediated depletion and LMX in terms of ERα degradation than Not-IDC cells (**Figures 3C, D**, respectively, **Supplementary Table 9**). Remarkably, also in this case, IDC cell lines belonging to the LumA class of the clinical surrogates appear more sensitive to GART inhibition-dependent receptor degradation (green dots in **Figures 3C, D**).

On this basis, we next evaluated the survival rate of women carrying LumA and LumB BC as a function of GART mRNA expression. According to the *in vitro* data, the RFS probability is significantly increased in women carrying a LumA breast tumor (i.e., ERα-positive, PR-positive/negative, HER2-negative) expressing low GART mRNA levels (**Figure 3E**; **Supplementary Table 4**) while no significant differences in the survival rate depending on GART expression have been evidenced for women with LumB tumors (i.e., ERα-positive, PR-positive/negative, HER2-positive) (**Figure 3F**; **Supplementary Table 4**)

Therefore, these data indicate the LumA ERα-expressing IDCs as the subclass of BC, which is more sensitive to GART inhibition.

### Validation of the impact of GART inhibition on ERα intracellular concentration and cell proliferation

**Figure 4:**
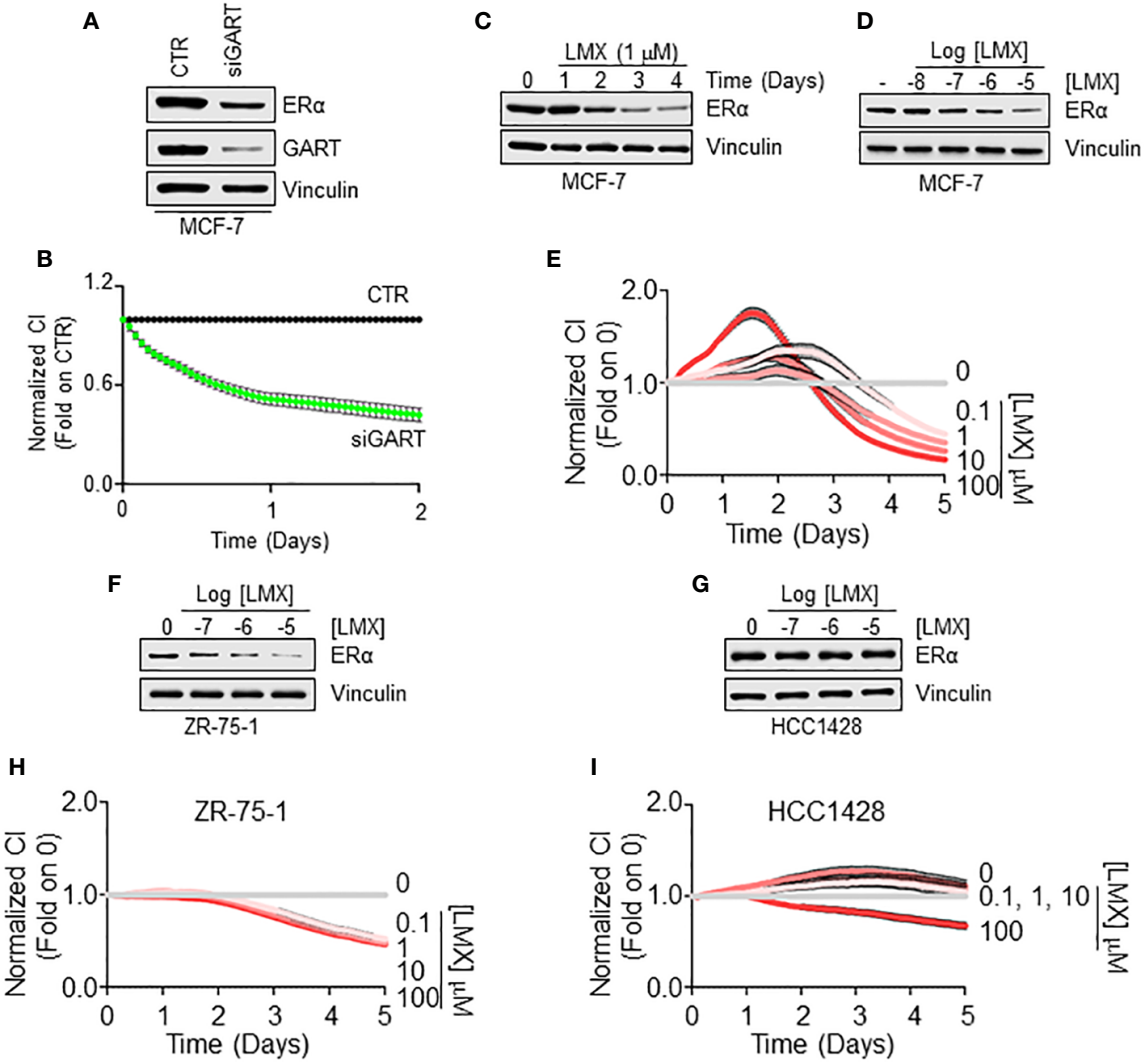
GART-dependent control of ERα stability and cell proliferation in Not-IDC and IDC breast cancer cells. Western blot analyses of ERα expression levels in MCF-7 cells treated with GART siRNA oligonucleotides for 24 hours **(A)** and with both the indicated time points **(C)** and the indicated doses **(D)** of lometrexol (LMX) doses for 48 hours. Blots are representative images of 4 independent experiments. Densitometric and statistical analyses are reported in **Supplementary Figure 1**. Growth curve analyses in MCF-7 cells were performed as indicated in the material and method section for 3 days in cells treated with GART siRNA oligonucleotides **(B)** and for 5 days in cells treated with the indicated doses of lometrexol (LMX) **(E)**. The graphs show the normalized cell index (*i.e.*, cell number), which is detected with the xCelligence DP device and calculated at each time point with respect to the control sample. Each sample was measured in quadruplicate. For details, please see the material and methods section. Western blot analyses of ERα expression levels in ZR-75-1 **(F)** and HCC1428 **(G)** cells treated at the indicated doses of lometrexol (LMX) doses for 48 hours. Blots are representative images of 4 independent experiments. Densitometric and statistical analyses are reported in **Supplementary Figure 2**. Growth curve analyses in ZR-75-1 **(H)** and HCC1428 **(I)** cells were performed as indicated in the material and method section for 5 days in cells treated with the indicated doses of lometrexol (LMX). The graphs show the normalized cell index (*i.e.*, cell number), which is detected with the xCelligence DP device and calculated at each time point with respect to the control sample. Each sample was measured in a quadruplicate. For details, please see the material and methods section.

To validate these observations, we next employed 3 ERα-expressing LumA BC cell lines, 2 of them belonging to the IDC histological type (i.e., MCF-7 and ZR-75-1 cells) and one of them being classified as Not-IDC (i.e., HCC1428) (21, 22). As shown in **Figures 4A, B** siRNA-mediated depletion of GART reduced both the total amount of ERα and the proliferation rate in MCF-7 cells. Accordingly, GART inhibition by LMX reduced both in a time and dose-dependent manner the ERα intracellular levels (**Figures 4C, D**; **Supplementary Figures 1A, B**) and the cell proliferation in MCF-7 cells (**Figure 4E**). Similar effects of LMX on ERα content and cell proliferation was scored also in the IDC ZR-75-1 cells (**Figures 4F, H**; **Supplementary Figure 2A**) but not in the Not-IDC HCC1428 cells (**Figures 4G, I**; **Supplementary Figure 2B**).

### The mechanism for the GART-dependent modulation of ERα intracellular levels

**Figure 5:**
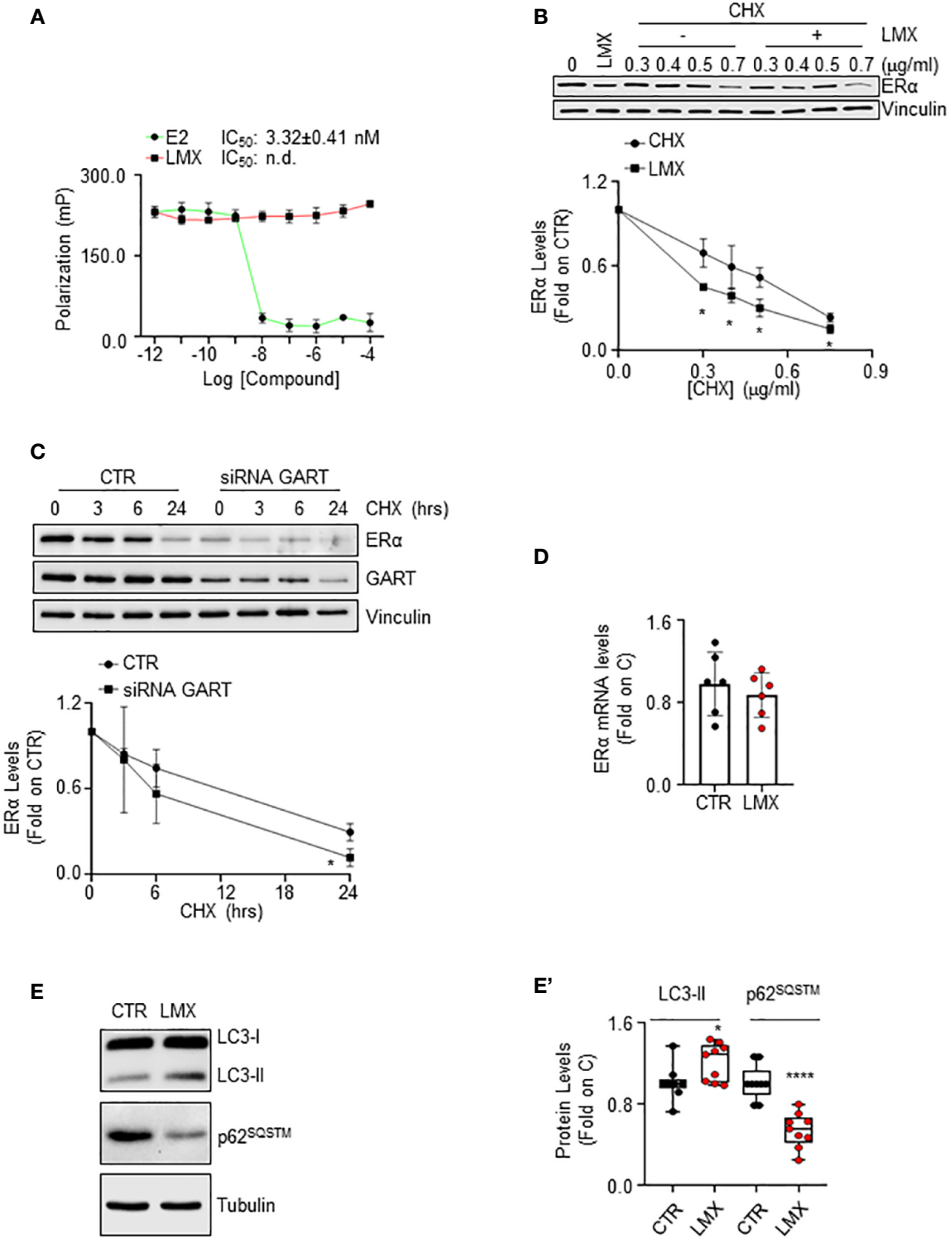
The mechanism through which GART controls ERα intracellular levels in IDC breast cancer cells. **(A)** *In vitro* ERα competitive binding assays for lometrexol (LMX), and 17β-estradiol (E2) were performed at different doses of the compounds and using a florescent E2 as the tracer. Relative inhibitor concentration 50 (IC_50_, *i.e.*, K_d_) is given in the graph. The experiment was performed twice in quintuplicate. **(B)** Western blot and relative densitometric analysis of ERα levels in MCF-7 cells pre-treated with cycloheximide (CHX) at the indicated doses for 6 hours and then treated with lometrexol (LMX - 1 µM) for 48 hours. The loading control was done by evaluating vinculin expression in the same filter. Blots are representative images of three independent experiments. Significant differences with respect to the CHX samples are calculated by Student t-test and indicated by * (*p* < 0.05). **(C)** Western blot and relative densitometric analysis of ERα and GART levels in MCF-7 cells treated with GART siRNA oligonucleotides for 24 hours and then treated at the indicated time points with cycloheximide (CHX – 1 µg/ml). The loading control was done by evaluating vinculin expression in the same filter. siRNA effectiveness has been evaluated in the same samples with GART antibody. Blots are representative images of three independent experiments. Significant differences with respect to the CTR samples are calculated by Student t-test and indicated by * (*p* < 0.05). **(D)** Real-time qPCR analysis of ERα mRNA levels in MCF-7 cells, treated for 48 hours with lometrexol (LMX – 1 µM). The experiment was repeated twice in triplicate. **(E)** Western blotting analysis and **(E’)** relative densitometric analyses of LC3 and p62^SQSTM^ cellular levels in MCF-7 cells treated lometrexol (LMX – 1 µM) for 48 hours. LC3 quantitation was performed using the formula LC3-II/(LC3-I+LC3-II). The loading control was done by evaluating tubulin expression in the same filter. Significant differences with respect to the CTR samples are calculated by Student t-test and indicated by * (*p* < 0.05) and **** (*p* < 0.0001).

ERα reduction in BC cells can be triggered by the direct ability of a ligand to bind the receptor and induce its degradation (40). Therefore, we performed ERα binding assays in the presence of different doses of LMX, and E2, to understand if LMX could bind the receptor *in vitro*. While E2 displaced the fluorescent E2 tracer with an IC_50_ (*i.e.*, K_d_) value of approximately 3.3 nM, as previously reported (13), LMX did not affect the ability of the recombinant ERα to bind the fluorescent E2 tracer at any of the tested LMX concentrations (**Figure 5A**).

LMX effect on ERα protein turnover rate was further tested in MCF-7 cells pre-treated with different doses of the protein synthesis inhibitor cycloheximide (CHX) for 6 hours before 48 hours of LMX administration. As expected, LMX and CHX reduced ERα intracellular content and LMX further diminished CHX-dependent reduction in the receptor intracellular amount (**Figure 5B**). Accordingly, time course analysis of CHX administration in MCF-7 cells shows that this protein synthesis inhibitor reduces ERα levels both in MCF-7 control cells and in MCF-7 cells where GART was transiently depleted (**Figure 5C**). Next, the ERα mRNA levels in MCF-7 cells, treated with LMX for 24 hours were measured, and found not to be modified by LMX (**Figure 5D**).

Therefore, LMX does not bind to ERα *in vitro* and does not change ERα mRNA levels, while it continues to induce receptor degradation in the presence of CHX and CHX has an increased effect in MCF-7 cells depleted of GART. Therefore, these results demonstrate that GART inhibition determines ERα degradation through post-translational mechanism not implying the binding of the inhibitor to the receptor.

It has been shown that purine-dependent starvation induces the activation of autophagy (41), and our laboratory provided evidence that ERα intracellular content in BC cells can be controlled by autophagy in addition to other cellular degradation mechanisms (42). Therefore, to investigate further the mechanistic connection between GART inhibition and the control of ERα intracellular levels, we hypothesized that LMX could induce the activation of autophagy. Consequently, we evaluated in cells treated with LMX for 48 hours both the cellular amount of LC3-II [*i.e.*, LC3-II/(LC3-I+LC3-II)], a marker of autophagosome number, and the levels of p62^SQSTM^, also known as sequestrosome, a substrate of autophagy that is considered as a marker of autophagolysosomes degradation activity (43). **Figures 5E, E’** show that LMX increased LC3-II cellular content while it decreased the p62^SQSTM^ intracellular levels. These data suggest that LMX increases both the autophagosome number and the autophagolysosome activity.

### The impact of the GART inhibition on E2:ERα signaling to cell proliferation

The ERα is a transcription factor, which regulates in an E2-dependent manner the expression of those genes that contain or not the estrogen response element (ERE) in their promoter region (40). In turn, we evaluated the impact of GART inhibition on the E2-dependent control of the expression of some ERE-containing (i.e., presenilin2 – pS2 and cathepsin D – Cat D) (16) and some non-ERE containing (i.e., cyclin D1 – CycD1 and Bcl-2) (44) genes in MCF-7 cells treated either with GART siRNA oligonucleotides or with LMX. The complete anti-estrogen fulvestrant (i.e., ICI182,780 - ICI) was also introduced as an internal control.

**Figure 6:**
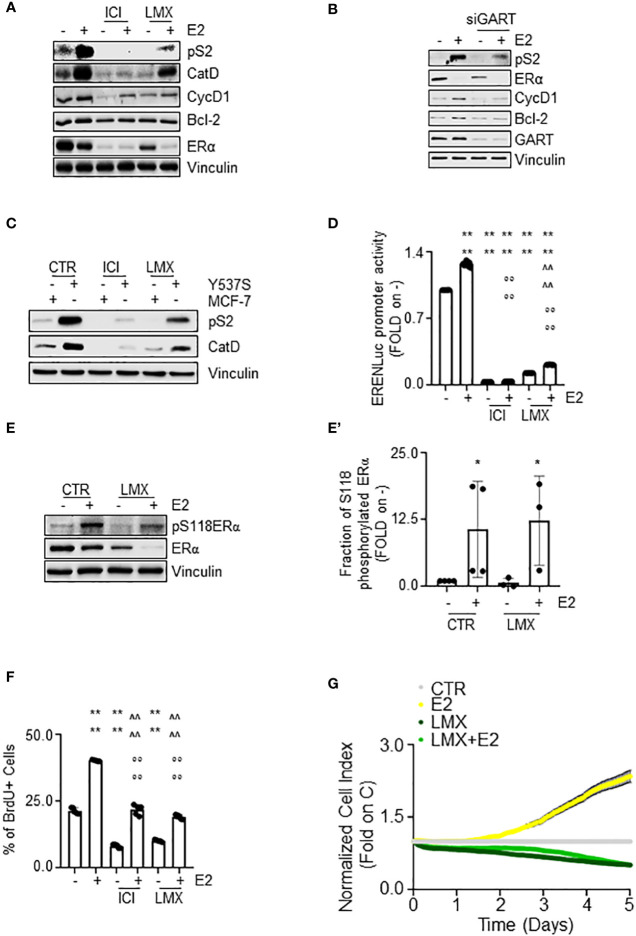
The impact of GART inhibition on E2:ERα signaling to cell proliferation. **(A)** Western blot of presenilin 2 (pS2), cathepsin D (CatD), cyclin D1 (CycD1), Bcl-2, and ERα expression levels in MCF-7 cells pre-treated with lometrexol (LMX 1 µM) for 48 hours and then treated for 24 hours with 17β-estradiol (E2 - 10 nM). The loading control was done by evaluating vinculin expression in the same filter. Panels show representative blots. Densitometric and statistical analyses are reported in **Supplementary Figures 3A–E**. **(B)** Western blot of presenilin 2 (pS2), cyclin D1 (CycD1), Bcl-2, and ERα expression levels in MCF-7 cells pre-treated with GART siRNA oligonucleotides for 24 hours and then treated for 24 hours with 17β-estradiol (E2 - 10 nM). The loading control was done by evaluating vinculin expression in the same filter. Panels show representative blots. Densitometric and statistical analyses are reported in **Supplementary Figures 3F–I**. **(C)** Western blot of presenilin 2 (pS2), and cathepsin D (CatD) in MCF-7 and Y537S cells treated with lometrexol (LMX 1 µM) for 48 hours. The loading control was done by evaluating vinculin expression in the same filter. Panels show representative blots. Densitometric and statistical analyses are reported in **Supplementary Figures 4A, B**. **(D)** Estrogen response element promoter activity in MCF-7 ERE-NLuc cells pre-treated with fulvestrant (ICI182,780 - ICI 100 nM) or lometrexol (LMX 1 µM) for 48 hours and then treated with 17β-estradiol (E2 10 nM) for additional 24 hours. The experiments were performed three times in quintuplicate. Significant differences were calculated with the Anova test. **** (*p-value* < 0.0001) indicates significant differences with respect to untreated (*i.e.*, -) sample. °°°° (*p-value* < 0.0001) indicates significant differences with respect to E2 treated sample. ^^^^ (*p-value* < 0.0001) indicates significant differences with respect to LMX treated sample. Western blot **(E)** and relative densitometric analyses **(E’)** of ERα and ERα S118 phosphorylation expression levels in MCF-7 cells pre-treated with lometrexol (LMX 1 µM) for 48 hours and then treated for 30 min with 17β-estradiol (E2 -10 nM). The loading control was done by evaluating vinculin expression in the same filter. Panels show representative blots of 4 independent experiments. Significant differences with respect to - sample are calculated by Student t-test and indicated by * *p*-value < 0.05. **(F)** Bromodeoxyuridine (BrdU) incorporation assay in MCF-7 cells treated with 17β-estradiol (E2 10 nM – 24 hours) after 48 hours pre-treatment with lometrexol (LMX 1 µM). The experiments have been performed twice in duplicate. Significant differences were calculated with the Anova test. **** (*p-value* < 0.0001) indicates significant differences with respect to untreated (*i.e.*, -) sample. °°°° (*p-value* < 0.0001) indicates significant differences with respect to E2 treated sample. ^^^^ (*p-value* < 0.0001) indicates significant differences with respect to LMX treated sample. **(G)** Real-time growth curves in MCF-7 cells treated with lometrexol (LMX 1 µM) in the absence and the presence of 17β-estradiol (E2 10 nM). The graphs show the normalized cell index (*i.e.*, cell number), which is detected with the xCelligence DP device and calculated at each time point with respect to the control sample. Each sample was measured in quadruplicate. For details, please see the material and methods section.

As shown in **Figure 6A**, pre-treatment of MCF-7 cells with LMX or ICI determined a reduction in the E2 ability to increase the expression of both pS2, CatD, CycD1, and Bcl-2 (**Figure 6A**; **Supplementary Figure 3A–D**). As expected, E2 induced also ERα degradation and ICI induced the same effect on receptor intracellular levels both in the presence or in the absence of E2, LMX was able to reduce the basal amount of ERα without blocking the E2 capability to trigger receptor degradation (**Figure 6A**; **Supplementary Figure 3E**). Accordingly, siRNA-mediated depletion of GART reduced the ERα intracellular content in MCF-7 cells but did not change the ability of E2 to further trigger the ERα reduction and prevented the E2-induced increase in pS2, CycD1 and Bcl-2 expression levels (**Figure 6B**; **Supplementary Figures 3F–I**). Next, we directly compared the ability of both ICI and LMX to reduce the expression levels of both pS2 and CatD in both MCF-7 and Y537S cells, which express a hyperactive receptor variant constitutively increasing receptor-dependent gene expression (23). As previously reported (23), the basal levels of both pS2 and CatD are increased in Y537S than in MCF-7 cells. Notably, both ICI and LMX reduced the pS2 and CatD protein abundance in Y537S cells (**Figure 6C**; **Supplementary Figures 4A, B**). Finally, we tested the ERα activity on a synthetic ERE-containing reporter gene stably transfected in MCF-7 cells (16) in the presence and the absence of both LMX, ICI, and E2. As expected (40), E2 increased the ERα transcriptional activity, which was completely prevented both in the presence and in the absence of ICI (**Figure 6D**). According to the data shown in **Figure 5A**, the pre-treatment of MCF-7 cells with LMX significantly reduced both the basal and the E2-induced ERα-mediated activation of the ERE-containing synthetic promoter but E2 was still significantly able to increase ERα transcriptional activity in the presence of the GART inhibitor (**Figure 6D**). Because the complete transcriptional activation of the ERα depends on the ability of E2 to trigger the receptor phosphorylation at the S118 site (40), we next treated MCF-7 cells with E2 after the administration of LMX and measured ERα S118 phosphorylation. **Figures 6E, E’** shows that LMX administration failed to block the E2-driven increase in the S118 phosphorylated ERα fraction in MCF-7 cells.

E2 is a complete mitogen for BC cells as it induces DNA synthesis, which results in cell cycle progression, and cell proliferation (40, 45). In turn, we next measured the effect of GART inhibition by LMX on E2-induced DNA synthesis by evaluating the bromodeoxyuridine (BrdU) incorporation in MCF-7 cells. Twenty-four hours of E2 administration increased the percentage of BrdU positive cells (**Figure 6F**) as previously reported (24). Notably, ICI and LMX administration reduced BrdU incorporation both in the absence and in the presence of E2 but did not block the ability of E2 to trigger BrdU incorporation (**Figure 6F**). Accordingly, E2 increased the cell number in a time-dependent manner (**Figure 6G**) and the co-treatment of MCF-7 cells with LMX prevented both the basal and the E2-induced time-dependent increase in cell number (**Figure 6G**).

Therefore, altogether these data indicate that GART inhibition i) decreases receptor transcriptional activity, but it does not impede the E2 functioning to regulate gene expression *via* the ERα and ii) deregulates the E2 ability to drive DNA synthesis and cell proliferation in MCF-7 cells.

### GART inhibition in association with 4OH-tamoxifen and CDK4/CDK6 inhibitor administration as a new treatment option for primary and MBC

**Figure 7:**
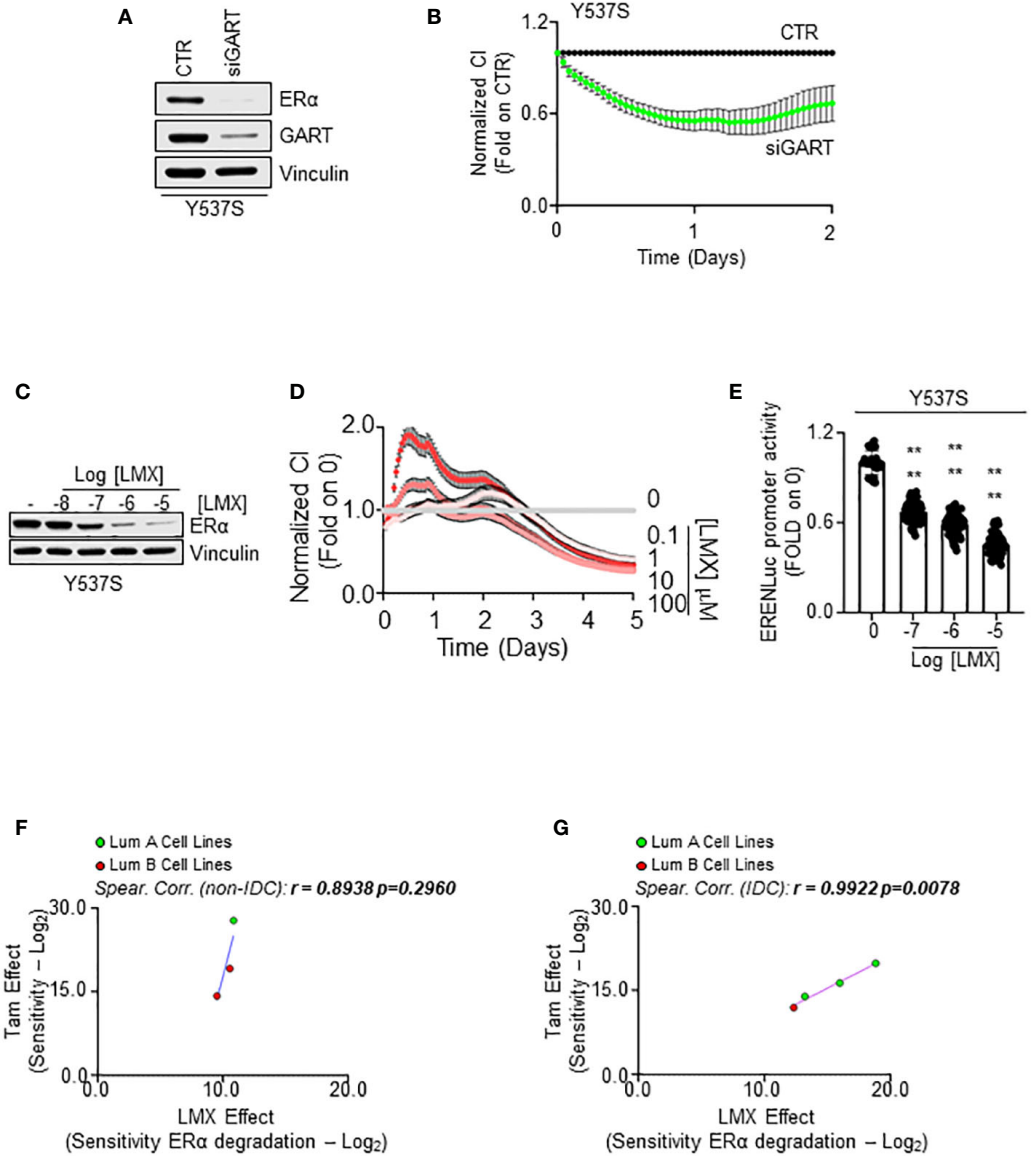
The impact of GART inhibition on 4OH-tamoxifen sensitive and resistant cell lines. Western blot analyses of ERα expression levels in Y537S cells treated with GART siRNA oligonucleotides for 24 hours **(A)** and with the indicated doses **(C)** of lometrexol (LMX) for 48 hours. Blots are representative images. Densitometric and statistical analyses are reported in **Supplementary Figure 4C**. Growth curve analyses in Y537S cells were performed as indicated in the material and method section for 3 days in cells treated with GART siRNA oligonucleotides **(B)** and for 5 days in cells treated with the indicated doses of lometrexol (LMX) **(D)**. The graphs show the normalized cell index (*i.e.*, cell number), which is detected with the xCelligence DP device and calculated at each time point with respect to the control sample. Each sample was measured in quadruplicate. For details, please see the material and methods section. **(E)** Estrogen response element promoter activity in MCF-Y537S ERE-NLuc cells treated with lometrexol (LMX 1 µM) for 48 hours. The experiments were performed three times in quintuplicate. Significant differences were calculated with the Anova test. **** (*p-value* < 0.0001) indicates significant differences with respect to untreated (*i.e.*, 0) sample. Linear regression and Spearman Correlation values between the sensitivity to 4OH-Tamoxifen (Tam) anti-proliferative activity calculated by performing the -Log_2_ transformation of the inhibitor concentration 50 (IC_50_) for Tam and the sensitivity to lometrexol (LMX)-induced ERα degradation effect as calculated by extrapolating the effective concentration 50 (EC_50_) and -Log_2_ transforming it in different BC cell lines stratified in luminal A (LumA, green dots) and luminal B (LumB, red dots) and non-invasive ductal carcinomas (Not-IDC) **(F)** or invasive ductal carcinomas (IDC) **(G)**. r and *p-values* are given in the main panels. Tam-induced dose-response curves are reported in **Supplementary Figure 5**.

In our initial screen, we identified GART as one of the 2 common proteins, which siRNA-mediated reduction induced ERα degradation and dampened cell proliferation in both MCF-7 and Y537S cells. Therefore, we next tested the impact of GART inhibition in Y537S cells. siRNA-mediated depletion of GART reduced both ERα levels and cell proliferation (**Figures 7A, B**, respectively) as well as LMX administration induced receptor degradation (**Figure 7C**; **Supplementary Figure 4C**) and an anti-proliferative effect (**Figure 7D**) in a dose-dependent manner.

To further evaluate if GART inhibition affects Y537S ERα transcriptional activity, we treated a Y537S cell line stably transfected with a synthetic ERE-containing reporter gene (26) with different doses of LMX for 48 hours. As shown in **Figure 7E**, the GART inhibitor significantly reduced in a dose-dependent manner the transcriptional activity of the Y537S mutant ERα.

Because Y537S cells are a well-known cell line modeling MBC that express a transcriptional hyperactive receptor point mutation, which confers resistance to ET drugs (e.g., Tam) (13, 23, 24, 46) and present results indicate that GART inhibition prevents the mutant receptor transcriptional activity and induces its degradation, we next decided to evaluate the possibility that GART inhibition could play a role in preventing the onset of resistance to ET and/or in inhibiting the proliferation of ET resistant cells.

Initial experiments were performed to understand if a correlation exists between the Tam and LMX effect in BC cells. For this purpose, growth curve analyses were done in the 7 above-mentioned BC cell lines (**Supplementary Table 9**) treated with different doses of Tam for 14 days (**Supplementary Figure 5A**). We calculated the IC_50_ for Tam in each cell line and then -Log_2_ transformed this value to have a measure of the sensitivity of the different BC cell lines to Tam. Comparison of the calculated sensitivity to Tam with that extrapolated by the DepMap portal in the same cell lines revealed a significant linear correlation between the two datasets (r = 0.7453 p=0.05) (**Supplementary Figure 5B**; **Supplementary Table 9**), thus confirming our cell lines to respond to Tam as expected.

Next, we compared the sensitivity to Tam with the sensitivity of the different cell lines to LMX-induced receptor degradation in all the tested cell lines and stratified the results according to both the histological type on the cell lines (i.e., IDC and Not-IDC) and the clinical surrogates of BC (i.e., LumA and Lum B). Interestingly, we found a linear correlation between the sensitivity to Tam and to the ability of LMX to determine a reduction in ERα intracellular content in both Not-IDC and IDC (**Figures 7F, G**; **Supplementary Table 9**) cell lines. However, this correlation was significant only in IDC cell lines (r=0.9922 p=0.0078). Notably, LumA cell lines appear to be the most sensitive to both drugs (green dots in **Figure 7G**).

Therefore, these data suggest that the combination of a drug inducing ERα degradation (i.e., LMX) with a drug blocking ERα transcriptional activities and functions (i.e., Tam) in LumA IDC could be an effective approach to treat this kind of breast tumor.

**Figure 8:**
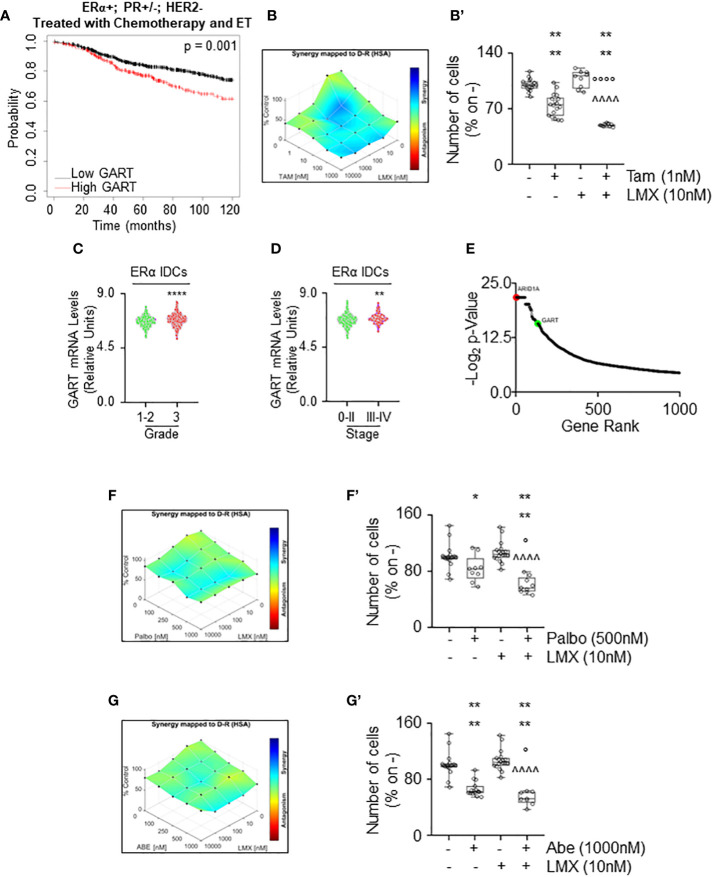
The synergy between lometrexol and clinically used drugs in 4OH-tamoxifen sensitive and resistant cell lines. **(A)** Kaplan-Meier plots showing the relapse-free survival (RFS) probability in women carrying breast tumors expressing ERα, both expressing or not progesterone receptor (PR) but not HER2 treated with chemotherapy and endocrine therapy (ET) as a function of GART mRNA levels. Significant differences between the RFS are given as *p*-value in each panel. Significant differences between the RFS are given as *p*-value in each panel. Data have been downloaded by the website (https://kmplot.com/analysis/). All possible cutoff values between the lower and upper quartiles are automatically computed (*i.e.*, auto-select best cutoff on the website), and the best performing threshold is used as a cutoff (35). **(B)** Synergy map of 12 days-treated MCF-7 cells with different doses of 4OH-Tamoxifen (Tam) and lometrexol (LMX). **(B’)** Growth curves in MCF-7 cells showing the synergic effect of each combination of compounds with selected doses. Significant differences have been calculated with the Anova test. **** (*p*-value < 0.0001) indicate significant differences with respect to untreated (*i.e.*, -,-) sample. °°°° (*p*-value < 0.0001) indicate significant differences with respect to Tam treated sample. ^^^^ (*p*-value < 0.0001) indicate significant differences with respect to LMX treated sample. GART mRNA levels in ERα-positive invasive ductal carcinomas (IDC) as a function of the grade **(C)** and the stage **(D)** of the tumor. **** (*p* < 0.0001) and ** (*p* < 0.01) indicate significant differences as calculated by the Student t-test. Data have been downloaded by the cBioPortal (https://www.cbioportal.org/) database. **(E)** Ranking of the genes in MCF-7 cells, which are important for the development of Tam resistance. Original data extrapolated by the **Supplementary Material** published in (47) have been used to generate the plot. ARID1A position (i.e., #2 red dot) was indicated as a positive control. GART position (i.e., #133) has been highlighted as a green dot. Synergy map of 7 days-treated Y537S cells with different doses of palbociclib (Palbo) **(F)** or of abemaciclib (Abe) **(G)** and lometrexol (LMX). **(F’, G’)** Growth curves in Y537S cells showing the synergic effect of each combination of compounds with selected doses. Significant differences have been calculated with the Anova test. **** (*p*-value < 0.0001) and * (*p*-value < 0.05) indicate significant differences with respect to untreated (*i.e.*, -,-) sample. ° (*p*-value < 0.05) indicates significant differences with respect to Palbo or Abe treated sample. ^^^^ (*p*-value < 0.0001) indicate significant differences with respect to LMX treated sample.

To test this hypothesis, we first evaluated the RFS rate as a function of GART mRNA expression of women with LumA BCs (i.e., ERα-positive, PR-positive/negative, HER2-negative) treated with chemotherapy and ET as we reasoned that this cohort of patients could benefit the most by the contemporary inhibition of both GART and ERα. Kaplan-Meier plot retrieved by the Kaplan-Meier Plotter database (https://kmplot.com/analysis/) (35) showed that in this group of patients a reduced expression of GART mRNA significantly prolongs the survival probability (**Figure 8A**; **Supplementary Table 4**). Prompted by these results we next performed proliferation studies in MCF-7 cells treated with different doses of LMX and Tam for 14 days and found significant synergy between these two drugs (**Figures 8B, B’**), thus sustaining that these inhibitors could be used in the combinatorial treatment of IDC LumA primary BCs.

Next, we sought to determine if GART could be considered also a target for the treatment of MBC. For this purpose, we measured the GART mRNA expression as a function of grade and stage of the tumor (8) as annotated in the Metabrick datasets extrapolated by the cBioPortal database (https://www.cbioportal.org/) (37, 38). In particular, we evaluated the mRNA expression of GART in ERα-positive IDCs. Interestingly, GART mRNA expression is increased both in grade 3 tumors with respect to grade 0-2 neoplasms (**Figure 8C**; **Supplementary Table 10**) and in BCs classified as stage III-IV with respect to stage 0-II tumors (**Figure 8D**; **Supplementary Table 10**). Moreover, CRISPR-CAS9 dropout screening performed in MCF-7 cells treated for 26 days with Tam (47) revealed that GART scored within the first 150 genes that are considered important in the development of Tam resistance in MCF-7 cells (**Figure 8E**; **Supplementary Table 10**). As a reference, the position of ARID1A, which has been demonstrated as a key gene for the onset of Tam resistance in MCF-7 cells (47) has been also shown in **Figure 8E**. Accordingly, GART mRNA is upregulated in MCF-7 cells resistant to Tam (48).

Because these data indicate that GART is highly expressed in ERα-positive IDCs, which are poorly differentiated and metastatic, and that this metabolic protein could play a role in the development of Tam resistance, GART could be considered also a target in BC cell lines resistant to ET. CDK4/CDK6 inhibitors (*i.e.*, palbociclib - Palbo and abemaciclib - Abe) treatment has been implemented in clinical practice as co-adjuvant drugs for ERα-expressing MBC management (5, 6, 8). Thus, we next treated Y537S cells with either Abe or Palbo in combination with LMX for 7 days. Results show that the proliferation of Y537S cells was synergistically reduced when CDK4/CDK6 inhibitors were co-administered with LMX (**Figures 8F, F’, G, G’**).

Overall, these data demonstrate that LMX exerts synergistic anti-proliferative activities with drugs being used for the treatment of primary and MBC.

### Evaluation of the antiproliferative effect of LMX in 3D models of primary and MBC

**Figure 9:**
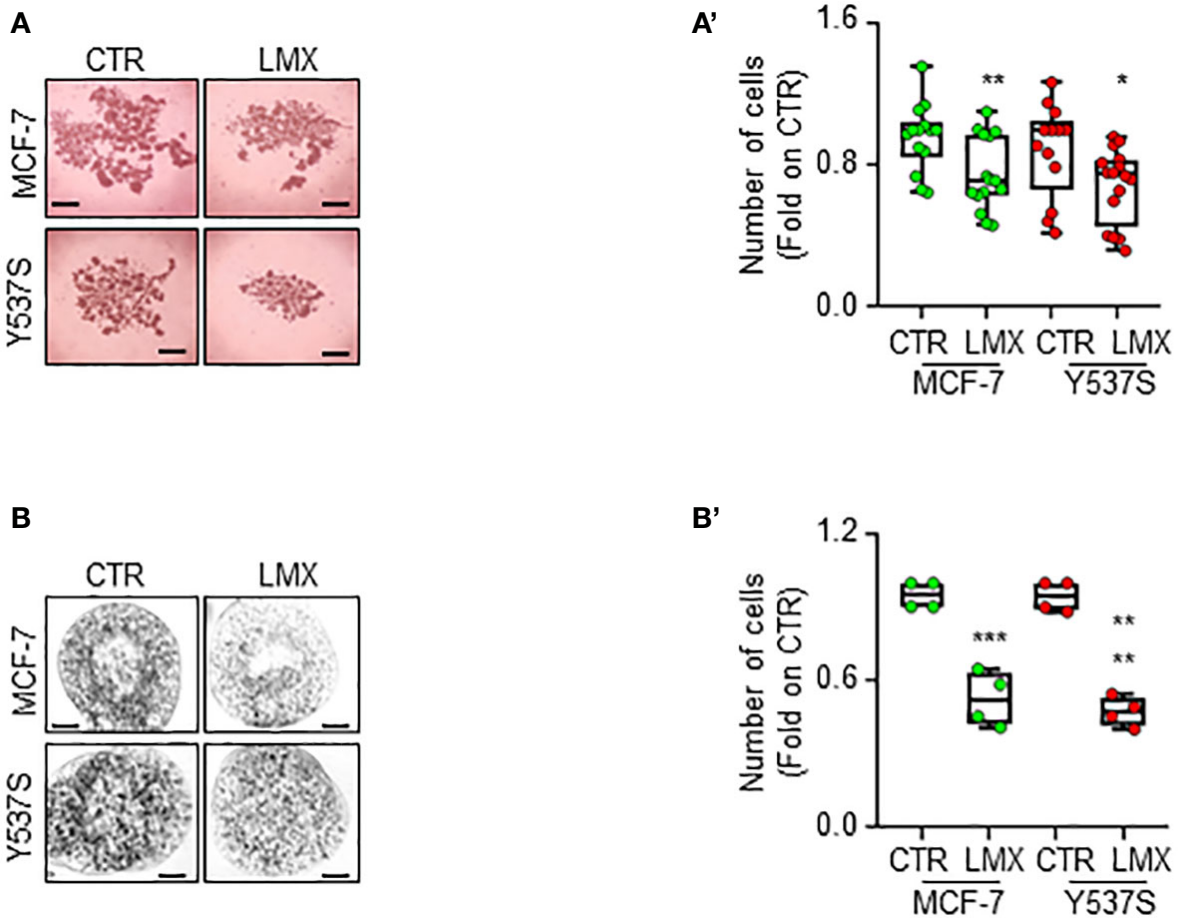
Effect of LMX in 3D-models of breast cancer. Images **(A, B)** and quantitation **(A’, B’)** of tumor spheroids surface area **(A, A’)** and alginate-based cultures **(B, B’)** generated in MCF-7 and Y537S cells, treated at time 0 with LMX (1 μM) or left untreated (CTR), for 7 days. The number of replicates is given as solid dots in the graphs. Significant differences with respect to the CTR sample were determined by unpaired two-tailed Student’s t-test: ** *p*-value < 0.01; * p-value<0.05. Scale bars equal to 50.0 mm.

Finally, we studied the anti-proliferative effects of LMX in MCF-7 and Y537S tumor cell spheroids as well as in alginate-based cultures (24) to understand if LMX could maintain its antiproliferative activity in cells grown in 3D structures (45). Tumor spheroids and cells included in alginate-based spheres grew in spheroids and inside alginate spheres within 7 days and LMX significantly prevented MCF-7 and Y537S cell proliferation both as spheroid (**Figures 9A, A’**) and in alginate-based cultures (**Figures 9B, B’**).

## Discussion

Metabolic reprogramming is a crucial aspect of cancer progression because, along with the deregulation of proliferative signaling, cancer cells adjust their metabolic pathways to sustain the energy and material demands of constant proliferation. However, such a significant reorganization exposes tumor cells to potential inhibition of the altered metabolic pathways, making it a promising avenue for novel cancer treatments. Targeting cancer cell metabolism has emerged as a new frontier in cancer management, as demonstrated by various studies (1–4). Notably, research has revealed that inhibiting certain metabolic proteins may induce ERα degradation and hinder the proliferation of BC cells (17–19). Therefore, the primary objective of this study was to identify metabolic proteins whose inhibition could impede BC cell proliferation by inducing ERα degradation.

By conducting a sequential functional genomic screen in a non-transformed breast cell line and two cell lines modeling primary and MBC, along with a metabolomic analysis in BC cells, we discovered that glutamine is overexpressed in ERα-positive breast cancer cells. Additionally, targeting GART, a molecule that regulates the initial substrate in the metabolic chain involving glutamine, induced ERα degradation and prevented tumor cell proliferation, without affecting the survival of non-transformed breast cells. Notably, both glutamine and GART are part of the *de novo* purine biosynthetic pathway (34).

The *de novo* purine biosynthesis pathway is crucial for mRNA synthesis and DNA replication in rapidly growing and replicating cancer cells, making it an attractive target for potential therapeutic interventions (4, 49). Analysis of GART expression in breast tumors revealed that this protein could serve as a promising pharmacological target in ERα-positive breast cancer cells, as patients with low GART mRNA levels have a higher chance of survival. Moreover, GART is overexpressed in breast tumors compared to normal breast epithelium. Interestingly, GART mRNA expression is lower in ERα-positive tumors than in ERα-negative ones, but higher in IDCs than in other histological tumor types. Tissue arrays confirmed that ERα-positive IDCs have higher GART protein levels than ERα-negative IDCs, and metabolomic analyses revealed that glutamine is more abundant in ERα-positive IDC cell lines and breast tumors than in ERα-negative ones. Inhibition of GART by siRNA-mediated depletion or lometrexol (LMX) inhibitor preferentially reduced cell proliferation and induced ERα degradation in ERα-positive IDC cell lines belonging to the LumA clinical surrogate class of breast cancer. Therefore, women with LumA tumors had significantly prolonged relapse-free survival rates if the tumor had low GART mRNA levels. However, in LumB tumors, women with high GART mRNA levels had higher survival rates than those with low GART mRNA expression, indicating that GART is not a suitable target for this type of breast cancer. Hence, the data suggest that personalized anti-tumor drug treatments should be used based on the specific subtype of the tumor to achieve the best results.

After evaluating the effect of GART inhibition in different ERα-positive BC cell lines, it was found that it only induces receptor degradation and prevents cell proliferation in ERα-positive LumA IDC cells. The reduction of ERα content in BC is an important treatment strategy as it leads to the inhibition of BC cell proliferation. Therefore, many novel SERDs are being developed with a safe pharmacological profile to treat this disease. A recent concept has been introduced where compounds that do not directly bind to ERα but induce its degradation through different cellular pathways can be used as ‘anti-estrogen-like’ compounds. Furthermore, because these compounds often target pathways not directly related to ERα, they have the advantage of being used in combination with classic ET drugs to achieve additional anti-tumor effects (11).

Interestingly, the reduction in ERα intracellular levels also determines the inhibition of E2 signaling to cell proliferation, thus demonstrating that GART inhibition can be considered a new strategy for primary BC treatment. We further studied the mechanism through which LMX determines ERα degradation and found that it does not bind to the receptor and does not modify the mRNA levels of the *ESR1* gene. Rather LMX acts at post-translational levels, as shown by the reported turnover studies and confirmed by the ability of CHX to work also under GART-depleted conditions. Because ERα intracellular content can be controlled by autophagy (42) and the purine-dependent cellular starvation determines the activation of autophagy (41), we investigated the details of the mechanistic connection between GART inhibition and ERα degradation by testing the ability of LMX to activate autophagy. Whitin the timeframe in which LMX induce receptor degradation, we observed that this GART inhibitor increases the number of autophagosomes and the autophagolysosome activity (as determined by LC3-II and p62^SQSTM^ intracellular amounts, respectively). Therefore, these observations indicate that the purine-dependent starvation determined by GART inhibition/depletion in BC cells induces the activation of autophagy, which in turn determines ERα degradation. Moreover, the fact that LMX reduces but does not block the ability of E2 to control receptor activities suggests that this inhibitor targets a pathway parallel to ERα signaling.

We also report that GART inhibition affects the stability and the activity of the Y537S ERα mutant as well as the proliferation of the Y537S cell line, which models MBC resistant to ET (23). This evidence opened the possibility that GART could be a target also in a MBC context. Accordingly, not only we noticed that ERα-positive LumA IDC cell lines, which are the more sensitive to the antiproliferative activity of Tam are also those cell lines in which the LMX-induced ERα degradation is higher, but also we report that the GART mRNA expression is high in ERα IDCs of high grade and stage and that women with LumA BC treated with both chemotherapy and ET have a higher survival rate when the tumor contains low GART mRNA levels. Moreover, GART has been reported as one of the most important genes involved in the development of Tam resistance and its expression is increased in Tam resistant MCF-7 cells (47, 48). Therefore, we conclude that GART inhibition could be exploited also to treat MBC and/or to try to avoid the development of ET resistance in primary tumors.

In support of this concept, we found that LMX displays a strong synergic effect when administered in combination with Tam in cells modeling primary tumors and with the CDK4/CDK6 inhibitors abemaiclib and palbociclib in cell lines modeling the MBC context resistant to Tam. Therefore, concomitant targeting of GART and ERα or GART and CDK4/CDK6 results in a synergistic anti-proliferative action of the administered drugs. Noteworthy, LMX still exerts its anti-proliferative activities in three-dimensional contexts of BC, which represent model systems closer to human tissues where the drug has to work (50).

The findings presented in this study have identified GART as a new metabolic target that can be used to hinder the growth of breast cancer cells, alongside other targets such as FASN, acid ceramidase, and DHFR (17–19). What is particularly noteworthy is that the data suggest that inhibiting GART could be an effective way to selectively impede the proliferation of IDCs and prevent the abnormal growth of MBC cells that express the hyperactive receptor Y537S ERα variant. To the best of our knowledge, these are novel findings that offer new avenues for treating primary and MBCs.

## Conclusions

In this study utilizing the GART inhibitor LMX (39) in various BC cell lines of different subtypes has revealed that LMX could act as an ‘anti-estrogen-like’ compound by inducing receptor degradation, and that the *de novo* purine biosynthetic pathway could be a promising target for treating LumA ERα-expressing primary and metastatic IDC. This indicates that selectively targeting tumor cell metabolism with metabolic pathway inhibitors could serve as additional drugs for ERα-expressing BC. In conclusion, we suggest that GART inhibition by LMX or other GART inhibitors could be an innovative and effective personalized treatment strategy for primary and MBC.

## Data availability statement

The original contributions presented in the study are included in the article/**Supplementary Material**. Further inquiries can be directed to the corresponding author.

## Author contributions

MC performed most of the experimental work. SL performed BrdU analyses. SB performed tumor spheroids experiments. FA conceptualized the research, formally analyzed the data, and wrote, reviewed, and edited the manuscript. All authors contributed to the article and approved the submitted version.

## Supplementary material

Supplementary Figure 1 Histograms relative to figures 4C, D. Densitometric analyses for figure 4C (A) and 4D (B). The experiments were performed 4 times. Significant differences were calculated with Anova test. **** (p value < 0.0001) and * (p value < 0.05) indicate significant differences with respect to untreated (i.e., 0) sample.

Supplementary Figure 2 The effect of lometrexol in ZR-75-1, HCC1428 cells and 3D models of breast cancer. **(A, B)** Densitometric analyses of the blots shown in figure 4F and 4G, respectively. The experiments were performed 4 times. Significant differences were calculated with Anova test. ** (p value < 0.01) indicate significant differences with respect to untreated (i.e., 0) sample.

Supplementary Figure 3 Histograms relative to figures 5A, B. Densitometric analyses for figure 5A **(A-E)** and for figure 5B **(F-I)**. The experiments were performed 4 times. Significant differences were calculated with Anova test. **** (p value < 0.0001), *** (p value < 0.001), ** (p value < 0.01) and * (p value < 0.05) indicate significant differences with respect to untreated (i.e., -) sample. °°°° (p value < 0.0001), °°° (p value < 0.001), °° (p value < 0.01) and ° (p value < 0.05) indicate significant differences with respect to E2 treated sample. ^^^^ (p value < 0.0001), ^^^ (p value < 0.001), and ^^ (p value < 0.01) indicate significant differences with respect to LMX treated sample.

Supplementary Figure 4 Histograms relative to figure 5C and 6C. Densitometric analyses for figure 5C **(A and B)** and for figure 6C **(C)**. The experiments were performed 4 times. Significant differences were calculated with Anova test. **** (p value < 0.0001), *** (p value < 0.001), ** (p value < 0.01) and * (p value < 0.05) indicate significant differences with respect to untreated Y537S samples (panel A and B) or untreated (i.e., 0) sample (C).

Supplementary Figure 5 4OH-Tamoxifen anti-proliferative effects in different BC cell lines. **(A)** Growth curve analyses in the indicated cell lines treated with the indicated doses of 4OH-Tamoxifen (Tam) for 12 days. **(B)** Linear regression and Spearman Correlation values between the sensitivity to Tam-induced anti-proliferative effect as downloaded by the DepMap portal (https://depmap.org/portal) and the calculated -Log2 transformed inhibitor concentration 50 (IC50) for Tam effects in the same BC cell lines. r and p-values are given in the main panels.

## Glossary

**Table:**

***Abe***	Abemaciclib
***AC***	adenocarcinoma
***AKT***	v-akt murine thymoma viral oncogene homolog 1 (AKT)
***BC***	breast cancer
***BrdU***	Bromodeoxyuridine
***CDK4***	cyclin-dependent kinase 4
***CDK6***	cyclin-dependent kinase 6
***CHX***	Cycloheximide
***DHFR***	dihydrofolate reductase
***DMEM***	Dulbecco’s Modified Eagle Medium
***E2***	17β-estradiol
***ERBB2***	V-Erb-B2 Avian erythroblastic Leukemia Viral Oncogene Homolog 2
***ERE***	estrogen-responsive element
***ERα***	estrogen receptor α
***ET***	Endocrine therapy
***FASN***	fatty acid synthase
***FDA***	Food and Drug Administration
***GART***	Phosphoribosylglycinamide Formyltransferase, Phosphoribosylglycinamide Synthetase, Phosphoribosylaminoimidazole Synthetase
***HER2***	Human Epidermal Growth Factor Receptor 2
***IDC***	invasive ductal carcinoma
***IGF1-R***	Insulin-Like Growth Factor 1 Receptor
***LC3***	microtubule associated protein 1 light chain 3 alpha
***LMX***	lometrexol
***LumA***	luminal A
***LumB***	luminal B
***MBC***	metastatic breast cancer
***Palbo***	Palbociclib
***PI***	Propidium Iodide
***PR***	progesterone receptor
***pS2***	presenilin2
***RFS***	relapse-free survival
***SQSTM***	sequestrosome
***Tam***	4OH-tamoxifen
***TFF1***	trefoil factor 1
***YY***	Buffer: Yoss Yarden Buffer
