# Supplementary material for: A functional genetic screen for metabolic proteins unveils GART and the de novo purine biosynthetic pathway as novel targets for the treatment of luminal A ERα expressing primary and metastatic invasive ductal carcinoma
Source: Front Endocrinol (Lausanne). 2023 Apr 18;14:1129162. doi: 10.3389/fendo.2023.1129162 (PMC10151738; doi:10.3389/fendo.2023.1129162)
Supplement: Supplementary Figure 1 — Histograms relative to figures 4C, D. Densitometric analyses for figure 4C (A) and 4D (B). The experiments were performed 4 times. Significant differences were calculated with Anova test. **** (p value < 0.0001) and * (p value < 0.05) indicate significant differences with respect to untreated (i.e., 0) sample. [file Presentation_1.pptx]

## Slide 1
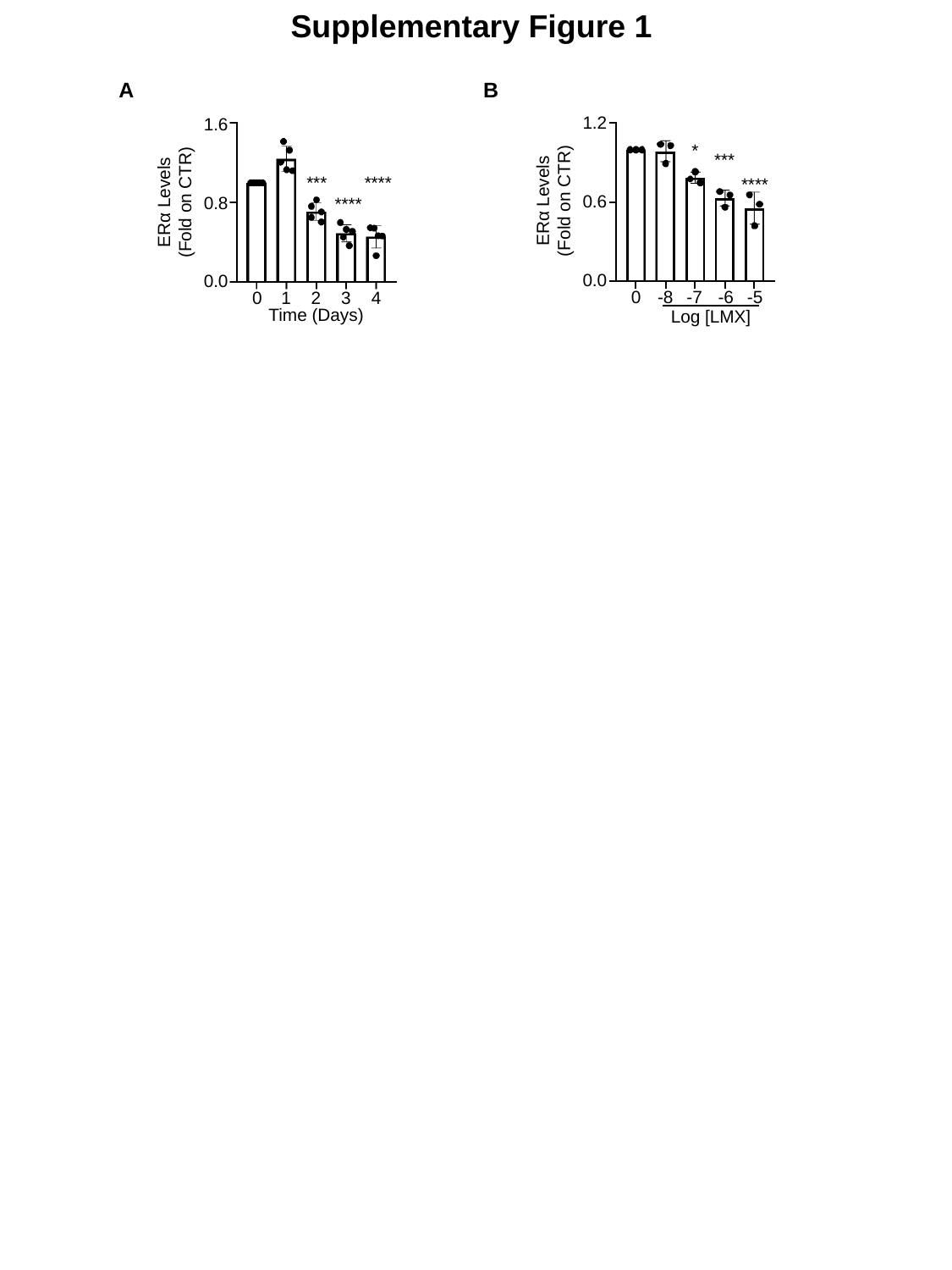

Supplementary Figure 1
A
B
1.2
ERα Levels
(Fold on CTR)
0.6
0.0
0
-8
-7
-6
-5
Log [LMX]
*
***
****
1.6
ERα Levels
(Fold on CTR)
0.8
0.0
0
1
2
3
4
Time (Days)
***
****
****

## Slide 2
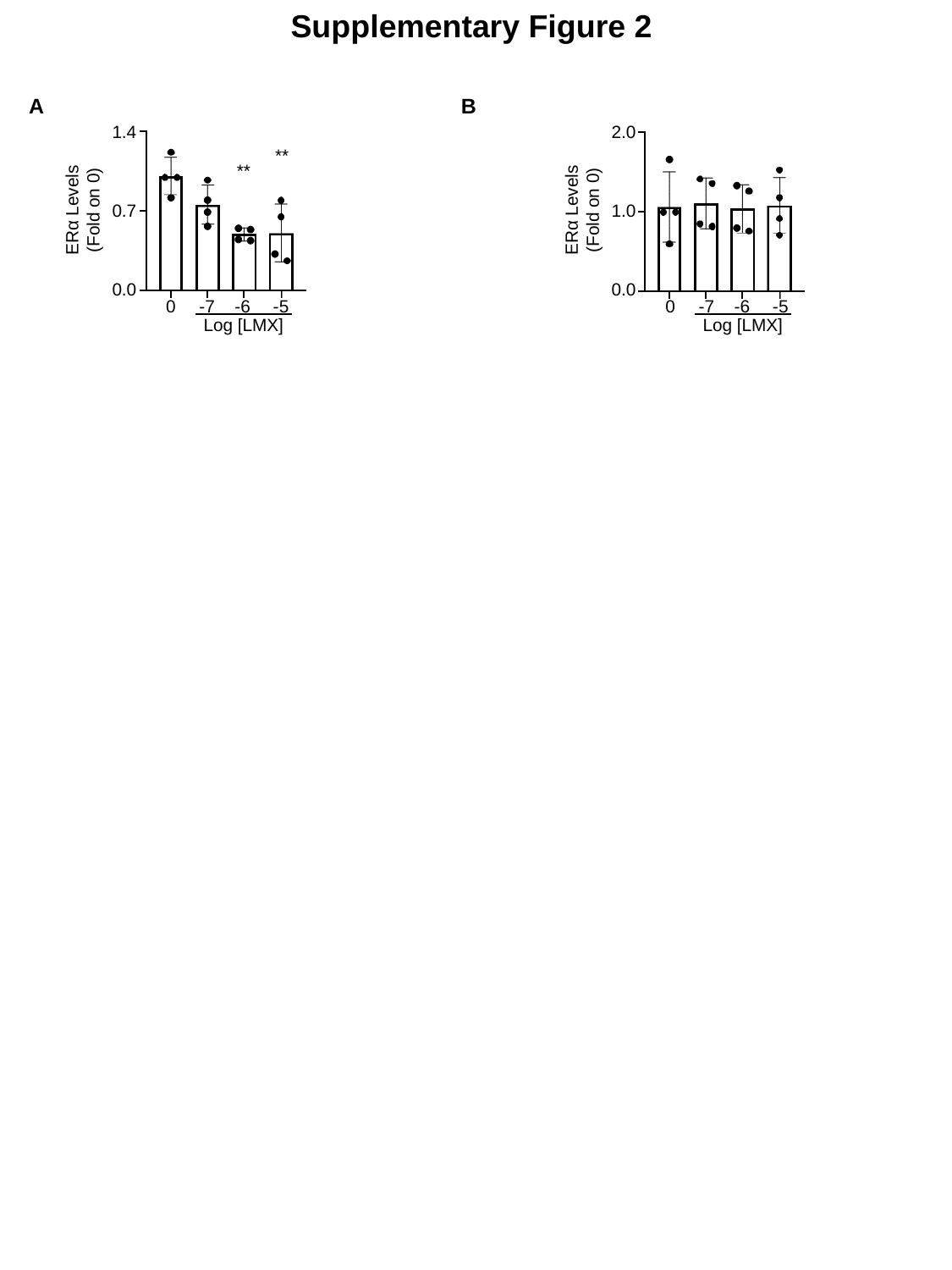

Supplementary Figure 2
A
B
1.4
**
**
ERα Levels
(Fold on 0)
0.7
0.0
0
-7
-6
-5
Log [LMX]
2.0
ERα Levels
(Fold on 0)
1.0
0.0
0
-7
-6
-5
Log [LMX]

## Slide 3
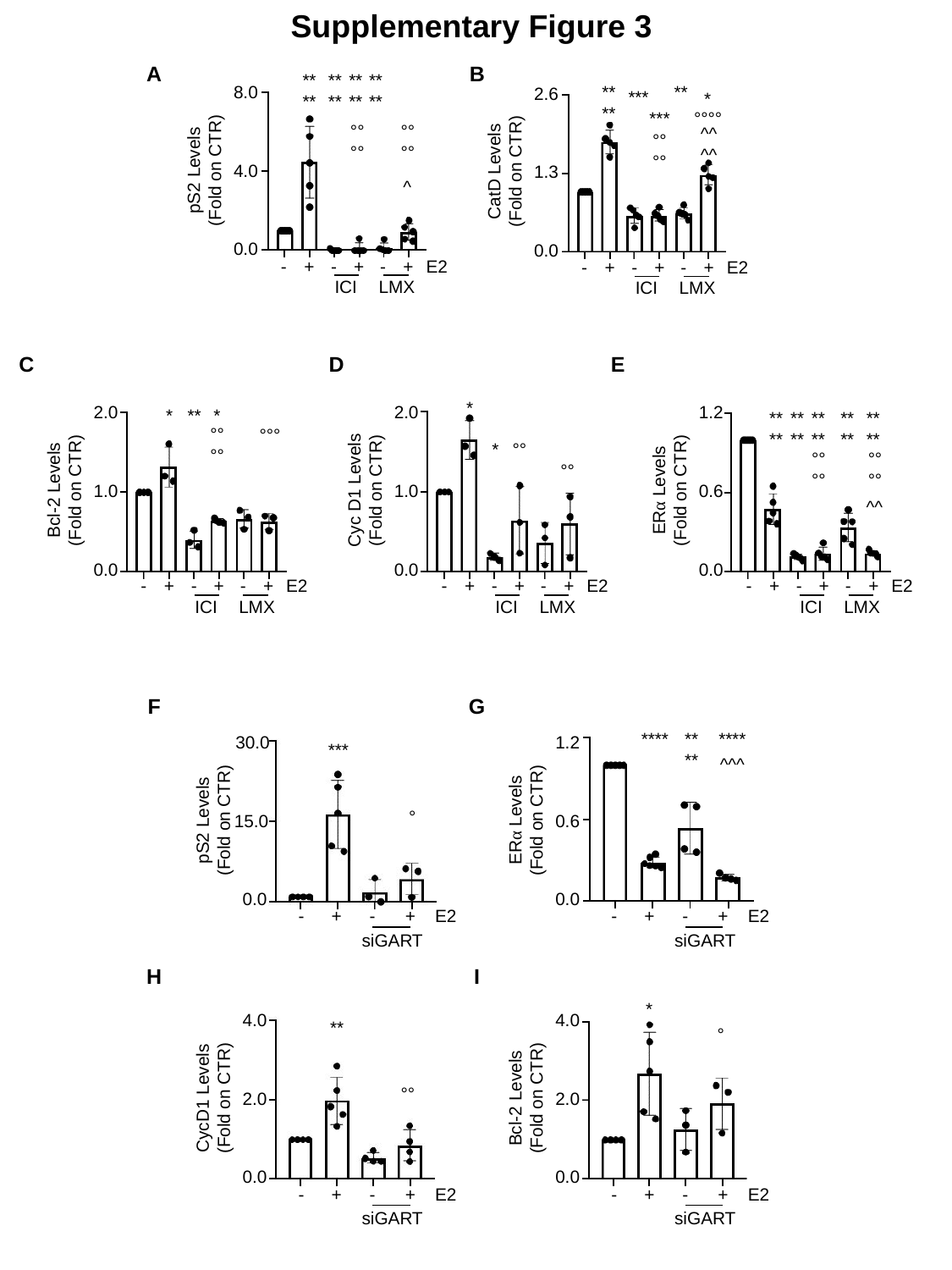

Supplementary Figure 3
A
B
**
**
**
**
**
**
**
**
8.0
pS2 Levels
(Fold on CTR)
4.0
0.0
E2
-
+
-
+
-
+
ICI
LMX
°°
°°
°°
°°
^
**
**
**
2.6
CatD Levels
(Fold on CTR)
1.3
0.0
E2
-
+
-
+
-
+
ICI
LMX
***
*
°°°°
***
^^
^^
°°
°°
C
D
E
*
2.0
Cyc D1 Levels
(Fold on CTR)
1.0
0.0
E2
-
+
-
+
-
+
ICI
LMX
*
°°
°°
2.0
Bcl-2 Levels
(Fold on CTR)
1.0
0.0
E2
-
+
-
+
-
+
ICI
LMX
*
**
*
°°
°°
°°°
1.2
ERα Levels
(Fold on CTR)
0.6
0.0
E2
-
+
-
+
-
+
ICI
LMX
**
**
**
**
**
**
**
**
**
**
°°
°°
°°
°°
^^
F
G
****
**
**
****
1.2
ERα Levels
(Fold on CTR)
0.6
0.0
E2
-
+
-
+
siGART
^^^
30.0
pS2 Levels
(Fold on CTR)
15.0
0.0
E2
-
+
-
+
siGART
***
°
H
I
*
4.0
Bcl-2 Levels
(Fold on CTR)
2.0
0.0
E2
-
+
-
+
siGART
°
4.0
CycD1 Levels
(Fold on CTR)
2.0
0.0
E2
-
+
-
+
siGART
**
°°

## Slide 4
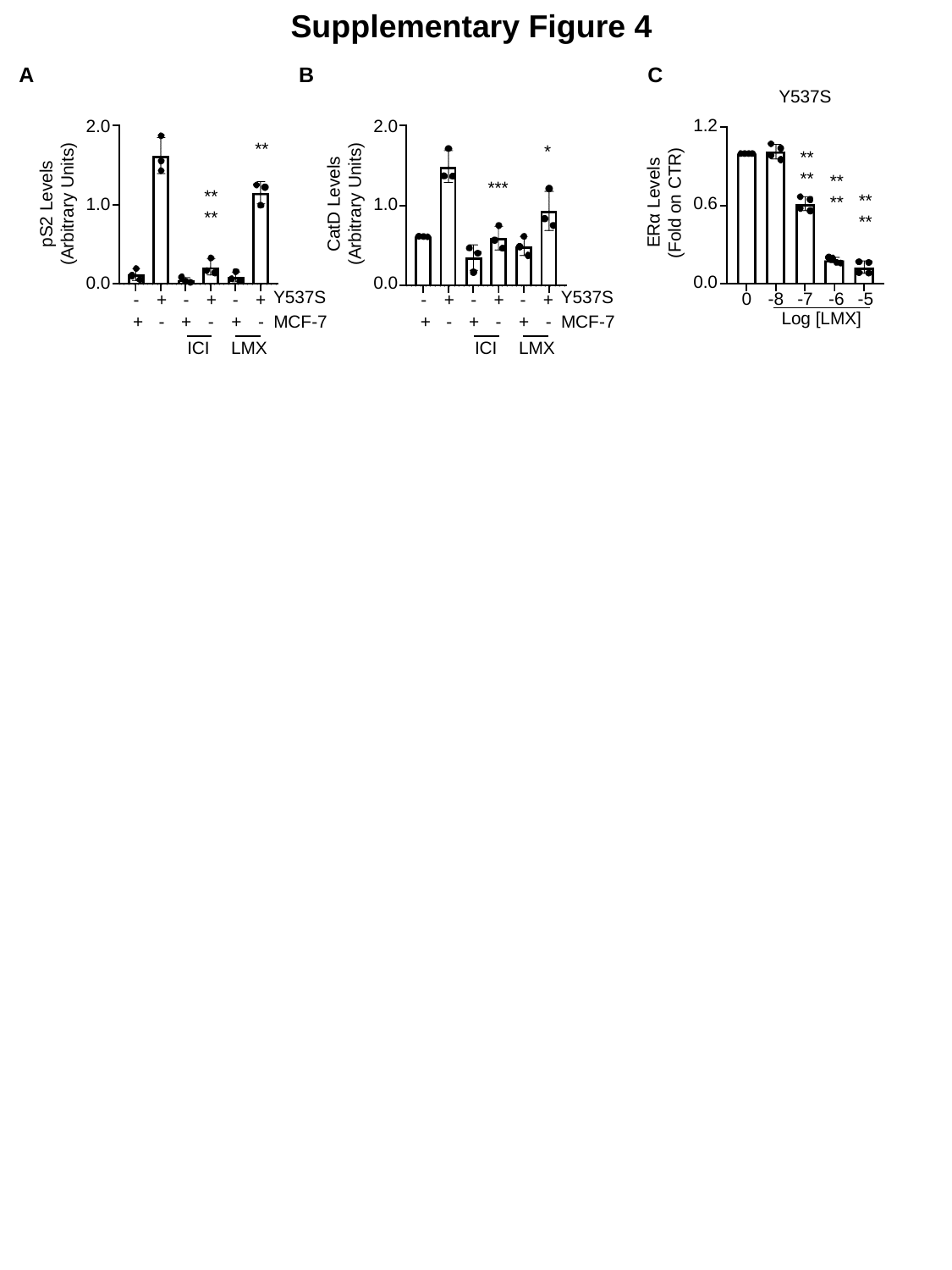

Supplementary Figure 4
A
B
C
Y537S
1.2
ERα Levels
(Fold on CTR)
0.6
0.0
0
-8
-7
-6
-5
Log [LMX]
**
**
**
**
**
**
2.0
pS2 Levels
(Arbitrary Units)
1.0
0.0
Y537S
-
+
-
+
-
+
+
-
+
-
+
-
MCF-7
ICI
LMX
**
**
**
2.0
CatD Levels
(Arbitrary Units)
1.0
0.0
Y537S
-
+
-
+
-
+
+
-
+
-
+
-
MCF-7
ICI
LMX
*
***

## Slide 5
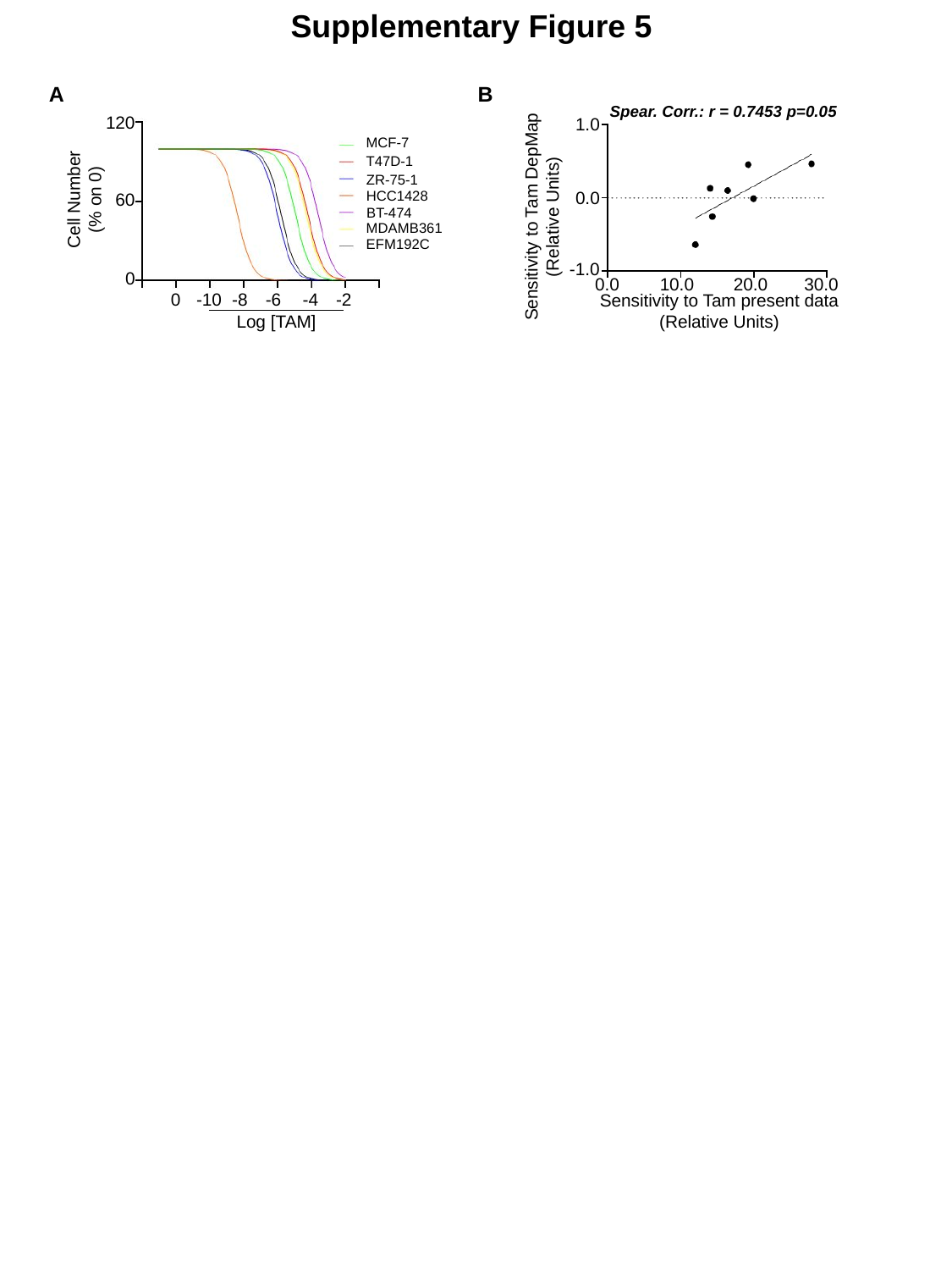

Supplementary Figure 5
A
B
Spear. Corr.: r = 0.7453 p=0.05
1.0
0.0
Sensitivity to Tam DepMap
(Relative Units)
-1.0
0.0
10.0
20.0
30.0
Sensitivity to Tam present data
(Relative Units)
120
MCF-7
T47D-1
ZR-75-1
HCC1428
BT-474
MDAMB361
EFM192C
Cell Number
(% on 0)
60
0
0
-10
-8
-6
-4
-2
Log [TAM]
